# A tandem Mannich addition–palladium catalyzed ring-closing route toward 4-substituted-3(2*H*)-furanones

**DOI:** 10.3762/bjoc.10.150

**Published:** 2014-06-27

**Authors:** Jubi John, Eliza Târcoveanu, Peter G Jones, Henning Hopf

**Affiliations:** 1 Institute für Organische Chemie, Technische Universität Braunschweig, Hagenring 30, D-38106 Braunschweig, Germany, Fax: +49 (0)531/391; 2 Institut für Anorganische und Analytische Chemie, Technische Universität Braunschweig, Hagenring 30, D-38106 Braunschweig, Germany

**Keywords:** aza-prostaglandin analogue, 3(2*H*)-furanone, Mannich addition, palladium catalysis, tandem reaction

## Abstract

A facile route towards highly functionalized 3(2*H*)-furanones via a sequential Mannich addition–palladium catalyzed ring closing has been elaborated. The reaction of 4-chloroacetoacetate esters with imines derived from aliphatic and aromatic aldehydes under palladium catalysis afforded 4-substituted furanones in good to excellent yields. 4-Hydrazino-3(2*H*)-furanones could also be synthesized from diazo esters in excellent yields by utilising the developed strategy. We could also efficiently transform the substituted furanones to aza-prostaglandin analogues.

## Introduction

Organic chemists welcome the introduction of facile tandem protocols because of the advantages of multiple bond formation in one-pot processes, which in turn makes the process economic and most importantly eco-friendly [1–2]. Conjugate addition is a very efficient tool used by synthetic chemists for the construction of carbon–carbon or carbon–heteroatom bonds [3]. Much effort has been invested to develop different variants of conjugate additions, both in catalyzed and non-catalyzed pathways. α-Halo ketones are known to react with zero-valent palladium like allyl halides to form oxa-π-allylpalladium complexes. Despite this fact, only minor attention has been paid to the formation of oxa-π-allylpalladium species from α-halo ketones and to their reactivity [4–8]. In this report we describe the use of a tandem methodology involving a Mannich addition/palladium-catalyzed ring-closing protocol for the facile synthesis of 4-substituted furanones.

**Figure 1:**
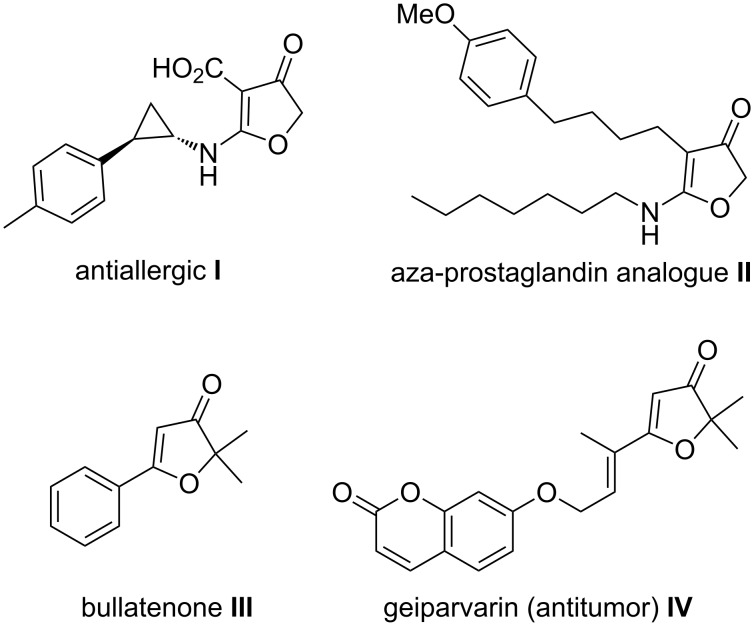
Bioactive molecules **I** [19], **II** [26], **III** & **IV** [21–22] with 3(2*H*)-furanone moiety.

Since 3(2*H*)-furanones form the main component of many natural products and pharmaceutically important compounds (Figure 1) a number of routes for their synthesis [9–18] to have been reported in the literature.

Substituted 3(2*H*)-furanones exhibit a wide range of biological activities such as antiallergic, antiulcer, antiinflammatory, and antitumor activities or selective COX-2 inhibition [19–26]. Synthetic routes for the preparation of these compounds involve transformations of substituted α-hydroxy-1,3-diketones [15,17], substituted furans [27–29], cyclization of allenic hydroxyketones [30], transition metal-catalyzed strategies (Au [31–33], Pt [34–35], Pd [36], Hg [37]), etc. Lately, organo-catalyzed asymmetric routes towards 3(2*H*)-furanones from 4-haloacetoacetates and nitrostyrene were reported by Lu et al. [38] and Yan et al. [39].

**Scheme 1:**
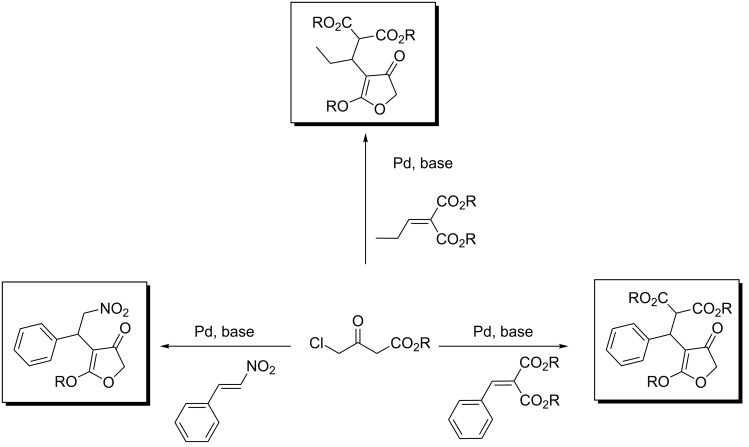
Pd-catalyzed synthesis of 3(*2H*)-furanones from activated alkenes [40].

We have recently reported on a palladium-catalyzed tandem methodology for the synthesis of 4-substituted-3(2*H*)-furanones from activated alkenes and 4-chloroacetoacetates (Scheme 1) [40].

The reaction was found to be general for a wide range of alkenes derived from aromatic and aliphatic aldehydes. The reaction proceeded via Michael addition of the acetoacetate to the alkene with a subsequent palladium-catalyzed ring closure of the primary adduct to form the furanone. In this paper, we report our investigations that extend the reaction to heteroatom-containing electrophiles such as imines and diazo esters.

## Results and Discussion

**Scheme 2:**
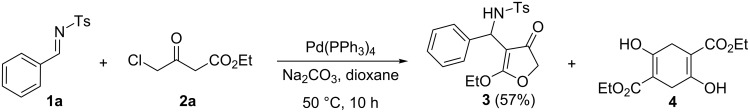
Pd-catalyzed synthesis of 3(*2H*)-furanone from tosylimine **1a**.

Following the work on nitrostyrenes, Yan et al. also reported a two-step asymmetric route towards 4-substituted-furanones from imines [41]. Recently Fructos et al. have shown that *N*-*p*-toluenesulfonyl-protected imines were better candidates for gold catalyzed Mannich addition of acetoacetates when compared to *N*-Boc (*tert*-butoxycarbonyl) and *N-*PMP (*p*-methoxyphenyl) imines [42]. Hence, we commenced our investigations with the reaction of tosylimine **1a** and ethyl 4-chloroacetoacetate (**2a**) in the presence of 5 mol % of Pd(PPh_3_)_4_ and 2.0 equivalents of Na_2_CO_3_, in dioxane at 50 °C for 10 hours. The reaction afforded 4-substituted-3(2*H*)-furanone **3** in 57% yield (Scheme 2). Interestingly, we could not detect the formation of diene-2,5-dicarboxylate **4** (formed by the dimerization of **2a**) as a side product, although this was observed previously in the reaction of activated alkenes [40] and 4-chloroacetoacetates.

Table 1 summarizes our efforts towards optimizing various reaction parameters with **1a** and **2a** as model substrates. Screening of bases revealed that Na_2_CO_3_ was more effective than either K_2_CO_3_ or KO*t*-Bu (Table 1, entries 1–3). When KO*t*-Bu was employed as base, byproduct **4** (formed by the dimerisation of **2a**) was formed in higher amounts (Table 1, entry 3). From the tested catalysts, Pd(PPh_3_)_4_ and Pd_2_dba_3_·CHCl_3_, and ligands, dppe and P(*o*-furyl)_3_, the combination of Pd_2_dba_3_·CHCl_3_ and dppe afforded the furanone **3** in 88% yield (Table 1, entry 8). A solvent screen revealed that dioxane was best for the present transformation from which the product **3** was obtained in 88% yield (Table 1, entries 8–10). It was found that lowering the temperature had a negative influence on the yield as **3** was obtained only in 43% even after 24 hours when the substrates **1a** and **2a** were stirred in the presence of Pd_2_dba_3_·CHCl_3_/dppe/Na_2_CO_3_ in dioxane at room temperature. When the catalyst loading was decreased to 1 mol %, the reaction afforded the furanone **3** in 78% yield after 10 hours (Table 1, entry 14). The catalytic role of palladium in the present transformation was proved by conducting two control experiments. The first reaction was performed only in the presence of base (Table 1, entry 12) and the second one only in the presence of the Pd_2_dba_3_·CHCl_3_/dppe combination (Table 1, entry 13). Furanone **3** was obtained in 65% yield from the first reaction whereas the second reaction afforded only trace amounts of the desired product. Thus we can see that under palladium catalysis there is an increase in the yield of 3(2*H*)-furanone **3** by 23% by comparing the first control experiment and the reaction depicted in entry 8 of Table 1, thereby proving that palladium is catalyzing the reaction.

**Table 1:** Optimisation studies^a^.

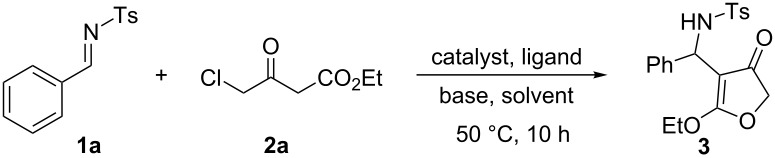				

Entry	Catalyst	Ligand	Base	Yield **3**^b^(%)

1	Pd(PPh_3_)_4_	–	Na_2_CO_3_	57
2	Pd(PPh_3_)_4_	–	K_2_CO_3_	34
3	Pd(PPh_3_)_4_	–	KO*t*-Bu	19^c^
4	Pd(PPh_3_)_4_	P(*o*-furyl)_3_	Na_2_CO_3_	66
5	Pd(PPh_3_)_4_	dppe	Na_2_CO_3_	70
6	Pd_2_dba_3_·CHCl_3_	–	Na_2_CO_3_	85
7	Pd_2_dba_3_·CHCl_3_	P(*o*-furyl)_3_	Na_2_CO_3_	82
8	**Pd_2_dba_3_·CHCl_3_**	**dppe**	**Na_2_CO_3_**	**88**
9	Pd_2_dba_3_·CHCl_3_	dppe	Na_2_CO_3_	80^d^
10	Pd_2_dba_3_·CHCl_3_	dppe	Na_2_CO_3_	84^e^
11	Pd_2_dba_3_·CHCl_3_	dppe	Na_2_CO_3_	43^f^
12	–	–	Na_2_CO_3_	65
13	Pd_2_dba_3_·CHCl_3_	dppe	–	trace
14	Pd_2_dba_3_·CHCl_3_	dppe	Na_2_CO_3_	78^g^

^a^Reaction conditions: **1a** (1.0 equiv), **2a** (1.1 equiv), catalyst (5 mol %), ligand (10 mol %), base (2.0 equiv), dioxane (2 mL), 50 °C, 10 h. ^b^Isolated yield. ^c^Dimerisation product **4** was formed in 27% yield.^d^CH_3_CN instead of dioxane. ^e^THF instead of dioxane. ^f^rt, 24 h. ^g^1 mol % of Pd_2_dba_3_·CHCl_3_, 5 mol % of dppe.

With optimal conditions in hand (imine (1.0 equiv), 4-chloroacetoacetate (1.1 equiv), Pd_2_dba_3_·CHCl_3_ (5 mol %), dppe (10 mol %) Na_2_CO_3_ (2.0 equiv) in dioxane at 50 °C for 10 h), studies towards the generality of the reaction were carried out with different imines **1a**–**c** derived from aromatic aldehydes with 4-chloro-acetoacetates **2a**,**b**.

**Figure 2:**
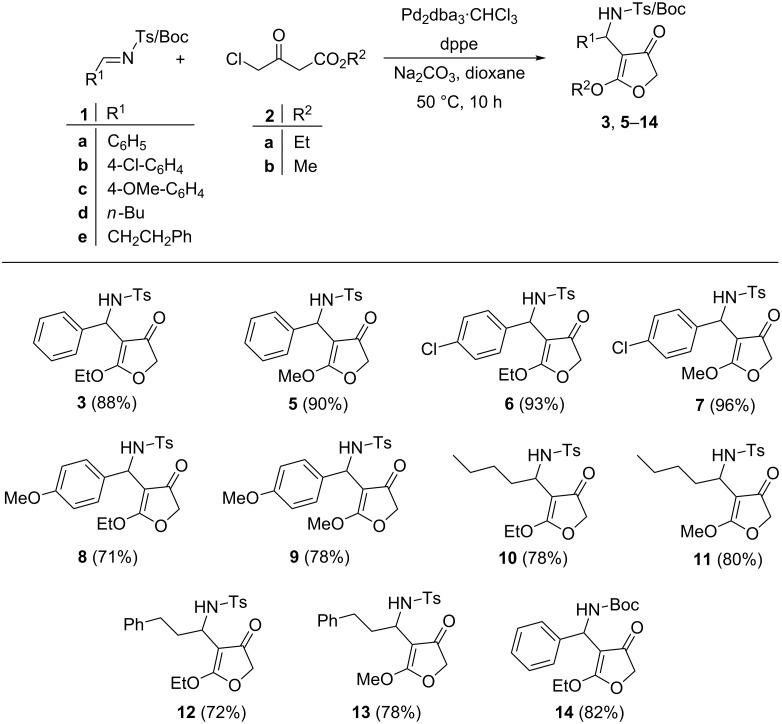
Generalisation with aromatic and aliphatic imines (reaction conditions: **1** (1.0 equiv), **2** (1.1 equiv), Pd_2_dba_3_·CHCl_3_ (5 mol %), dppe (10 mol %), Na_2_CO_3_ (2.0 equiv), dioxane (2 mL), 50 °C, 10 h, isolated yield).

**Figure 3:**
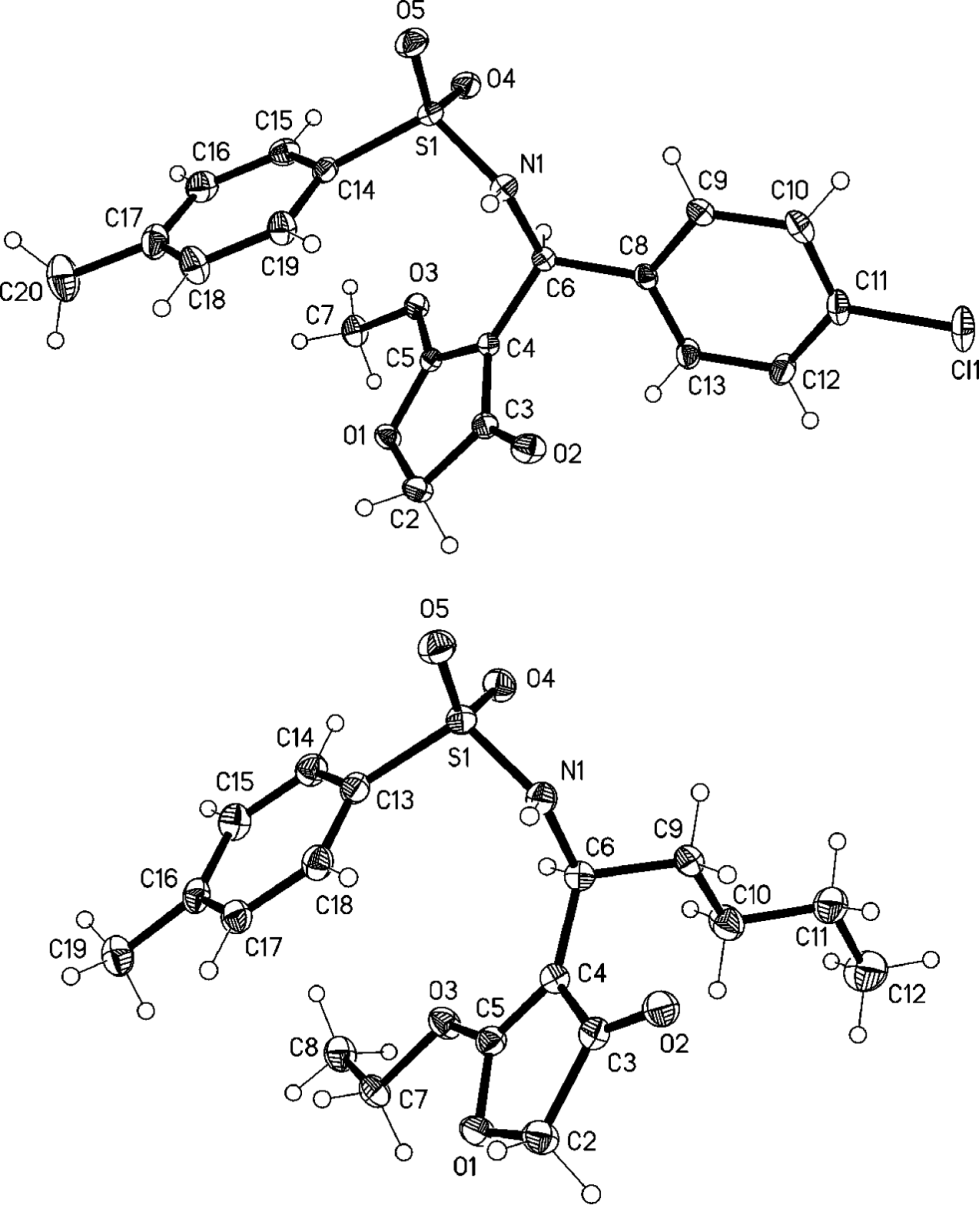
Thermal ellipsoid diagrams (50% probability levels) of 4-substituted-3(2*H*)-furanones **7** (above) and **10** (below).

In all the cases substituted 3(2*H*)-furanones were obtained in good to excellent yields (Figure 2). The reaction was also extended to tosylimines derived from aliphatic aldehydes, pentanal (for **1d**) and 3-phenylpropionaldehyde (for **1e**) and thus we could rule out any influence of the aromatic moiety on the outcome of the reaction. The corresponding 4-susbtituted-3(2*H*)-furanones were obtained from these two substrates in good yields (Figure 2). We also tried a reaction with *N*-Boc protected imine under optimized conditions and the corresponding furanone was obtained in good yield (Figure 2, compound **14**). The structure elucidation of the furanone products **3**, and **5**–**14** was accomplished by the usual spectroscopic methods (see Supporting Information File 1) and also by X-ray structure determination [43] for furanones **7** (product formed by the reaction of **1b** with **2b**) and **10** (product formed by the reaction of **1d** with **2a**) (Figure 3).

We believe that the mechanism of the present furanone synthesis from imines is similar to that reported for activated alkenes [40]. This tandem protocol has two steps, the first being a Mannich addition of the enolate to the imine. To find out whether Mannich addition is palladium catalyzed, we carried out two reactions of tosylimine **1a** with methyl acetoacetate. The first transformation was carried out under basic conditions (Na_2_CO_3_ (1.0 equiv), dioxane, 50 °C, 6 h) and the second under optimized conditions. Both reactions afforded the Mannich addition product in similar yields, proving that Mannich addition was uncatalyzed.

**Scheme 3:**
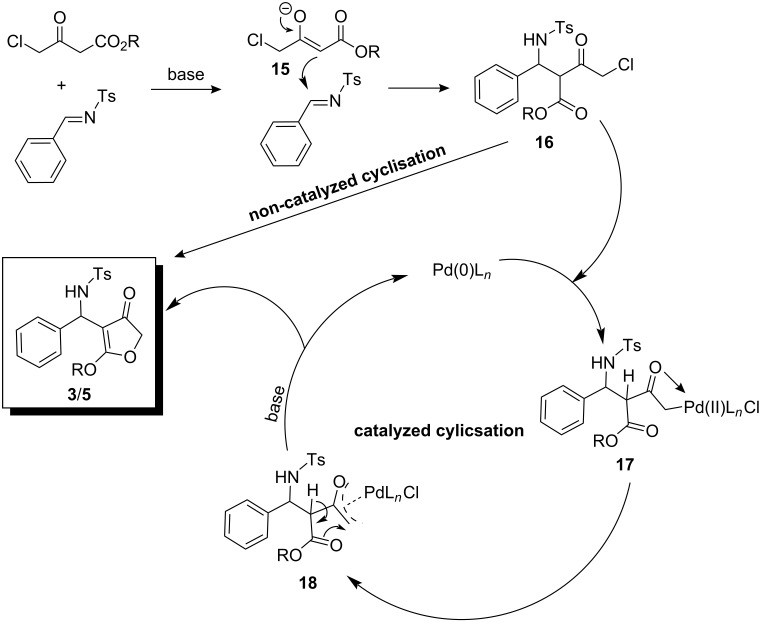
Mechanism of formation of the 3(2*H*)-furanone derivative from an imine.

Based on these results, we propose a plausible mechanism for 3(2*H*)-furanone synthesis from tosylimines and 4-chloroacetoacetate (Scheme 3). The reaction starts with the Mannich addition of the enolate **15** to the carbon atom of the imine double bond to form intermediate **16**. This step is the same for both the catalyzed and the uncatalyzed pathway. The second step of the catalyzed route involves the oxidative addition of Pd(0)L*_n_* to the C–Cl bond of Mannich adduct **16** to form **17**. The oxy-π-allylpalladium intermediate **18** can then be formed from intermediate **17** [4–8]. The final step of the catalyzed mechanism, i.e., the ring closure towards the formation of 3(2*H*)-furanone is initiated by the abstraction of the acidic proton by the base and consequent ester enolate attack to the end carbon of the oxy-π-allylpalladium intermediate.

**Scheme 4:**
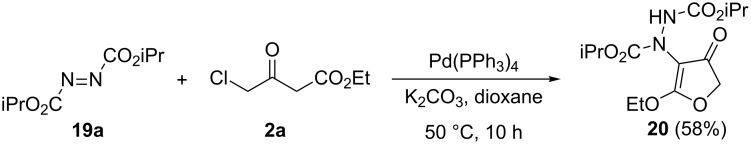
Pd-catalyzed synthesis of 3(*2H*)-furanone from diazoester **19a**.

Having established an efficient method for the synthesis of highly functionalized furanones from imines, we were interested next in extending the range of the electrophiles by introducing different functionalities at the 4-position of the 3(2*H*)-furanone. We chose to check the reactivity of diisopropylazodicarboxylate (**19a**) with ethyl 4-chloroacetoacetate (**2a**) under palladium catalysis. In the initial reaction, **19a** was treated with **2a** in the presence of Pd(PPh_3_)_4_ as catalyst and base (K_2_CO_3_) in dioxane at 50 °C. After 10 hours, the expected product 4-hydrazino-3(2*H*)-furanone **20** was isolated in 58% yield (Scheme 4).

Table 2 describes the screening of different reaction conditions to improve the yield of **20**. First we tested the efficacy of different bases K_2_CO_3_, Na_2_CO_3_ and KO*t*-Bu, of which Na_2_CO_3_ proved to be the best (Table 2, entries 1–3). The highest yield for 4-hydrazino-3(2*H*)-furanone was given when Pd(PPh_3_)_4_ alone was used among different catalyst/ligand combinations tested with Pd(PPh_3_)_4_ and Pd_2_dba_3_^.^CHCl_3_ as catalysts and dppe and P(*o*-furyl)_3_ as ligands (Table 2, entries 2, and 4–8). The product was obtained in better yields when dioxane was used as the solvent (Table 2, entries 2, 9 and 10). At room temperature the reaction with optimised catalyst/solvent system furnished only 38% of the substituted furanone even after 24 hours (Table 2, entry 11). A control experiment was done without the catalyst/ligand combination but only in the presence of base, which afforded the furanone in 69% yield (Table 2, entry 12). Thus by comparing the control experiment and the reaction depicted in entry 2 of Table 2 we can confirm that palladium catalyzes the reaction.

**Table 2:** Optimisation studies^a^.

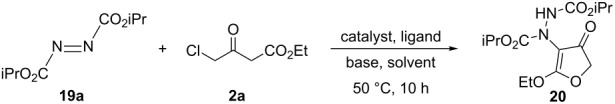				

Entry	Catalyst	Ligand	Base	Yield **20**^b^(%)

1	Pd(PPh_3_)_4_	–	K_2_CO_3_	58
**2**	**Pd(PPh_3_)_4_**	**–**	**Na_2_CO_3_**	**90**
3	Pd(PPh_3_)_4_	–	KO*t*-Bu	35^c^
4	Pd(PPh_3_)_4_	P(*o*-furyl)_3_	Na_2_CO_3_	75
5	Pd(PPh_3_)_4_	dppe	Na_2_CO_3_	79
6	Pd_2_dba_3_^.^CHCl_3_	–	Na_2_CO_3_	83
7	Pd_2_dba_3_^.^CHCl_3_	P(*o*-furyl)_3_	Na_2_CO_3_	80
8	Pd_2_dba_3_^.^CHCl_3_	dppe	Na_2_CO_3_	76
9	Pd(PPh_3_)_4_	–	Na_2_CO_3_	58^d^
10	Pd(PPh_3_)_4_	–	Na_2_CO_3_	82^e^
11	Pd(PPh_3_)_4_	–	Na_2_CO_3_	38^f^
12	–	–	Na_2_CO_3_	69

^a^Reaction conditions: **19a** (1.0 equiv), **2a** (1.1 equiv), catalyst (5 mol %), ligand (10 mol %), base (2.0 equiv), dioxane (2 mL), 50 °C, 10 h. ^b^Isolated yield. ^c^Dimerization product **4** was formed in 23% yield. ^d^CH_3_CN instead of dioxane. ^e^THF instead of dioxane. ^f^rt, 24 h.

**Figure 4:**
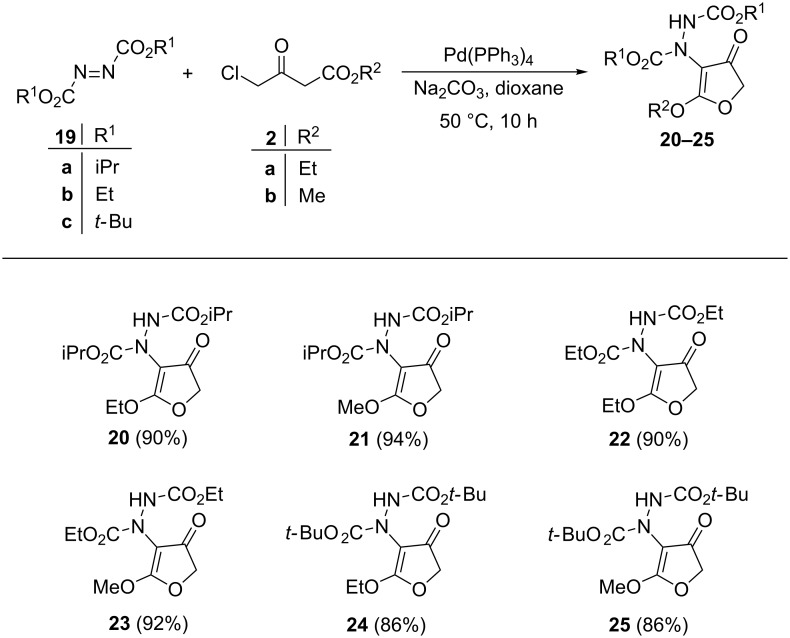
Generalisation with diazo esters (reaction conditions: **19** (1.0 equiv), **2** (1.1 equiv), Pd(PPh_3_)_4_ (5 mol %), Na_2_CO_3_ (2.0 equiv), dioxane (2 mL), 50 °C, 10 h, isolated yield).

The generality of the reaction was checked with different diazo esters **19a**–**c** with 4-chloroacetoacetate esters **2a** and **2b** under the optimized conditions (diazo ester (1.0 equiv), 4-chloroacetoacetate (1.1 equiv), Pd(PPh_3_)_4_ (5 mol %), Na_2_CO_3_ (2.0 equiv) in dioxane at 50 °C for 10 h). All reactions afforded the corresponding 4-hydrazino-3(2*H*)-furanones in excellent yields (Figure 4).

**Scheme 5:**
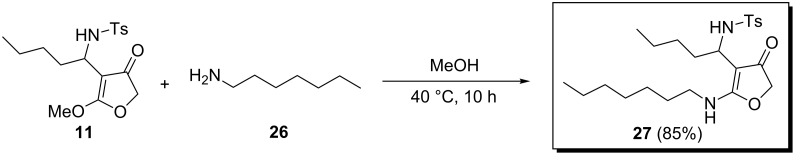
Synthesis of aza-prostaglandin analogue.

The possibility for further functionalization of 4-substitued furanones was checked by treating **11** with *n*-heptylamine in MeOH at 40 °C. After 10 hours the aza-prostaglandin analogue [26] **27** was isolated in 85% yield (Scheme 5).

## Conclusion

We have developed an efficient protocol for the synthesis of 4-substituted 3(2*H*)-furanones by the reaction of imines or diazo esters with 4-chloroacetoacetates under palladium catalysis. We could extend the reaction to imines derived from aromatic and aliphatic aldehydes. The reaction proceeded via a catalyzed tandem Mannich addition–palladium-catalyzed ring-closing pathway to afford various 3(2*H*)-furanones in good to excellent yields. We could further apply this route to the preparation of an aza-prostaglandin analogue. The synthesized molecules are currently being screened for biological activities. We have also extended the reaction to triple-bonded electrophiles such as acetylenes, benzyne and nitriles; the results will be reported in due course. Studies are in progress to develop a stereoselective version of the process.

## Supporting Information

File 1 Experimental part and NMR spectra.
