# Supplementary material for: A tandem Mannich addition–palladium catalyzed ring-closing route toward 4-substituted-3(2H)-furanones
Source: Beilstein J Org Chem. 2014 Jun 27;10:1462–70. doi: 10.3762/bjoc.10.150 (PMC4077403; doi:10.3762/bjoc.10.150)

Supporting Information

for

A tandem Mannich addition–palladium catalyzed ring-closing route toward 4-substituted-3(2*H*)-furanones

Jubi John[1], Eliza Târcoveanu[1], Peter G. Jones[2] and Henning Hopf*[1]

Address: [1]Institute für Organische Chemie, Technische Universität Braunschweig, Hagenring 30, D-38106 Braunschweig, Germany, Fax: +49 (0)531/391 and [2]Institut für Anorganische und Analytische Chemie, Technische Universität Braunschweig, Hagenring 30, D-38106 Braunschweig, Germany.

Email: Henning Hopf* - h.hopf@tu-bs.de

* Corresponding author

Experimental part and NMR spectra

Table of Contents

General methods	S2
Experimental procedures & compound characterisation	S2–S7
X-ray structure determination	S7–S8
^{1}H NMR & ^{13}C NMR spectra	S9–S26

General methods

All chemicals were of the best grade commercially available and were used without further purification. Imines **1a**–**c** were purchased from Sigma Aldrich. 4-Chloroacetoacetates **2a,b** and azoesters **19a**–**c** were purchased from Acros Organics. All solvents were purified according to standard procedures; dry solvents were obtained according to the literature methods and stored over molecular sieves. Analytical thin layer chromatography was performed on polyester sheets pre-coated with silica gel containing fluorescent indicator (POLYGRAM® SIL G/UV$_{254}$). Gravity column chromatography was performed using silica gel, and mixtures of pentane/ethyl acetate were used for elution. Melting points were measured with a Büchi 530 melting point apparatus and are uncorrected. IR spectra were recorded with a Bruker Tensor 27 spectrometer. NMR spectra were recorded with Bruker DRX-400 (400.1 MHz for ^{1}H NMR, 100.6 MHz for ^{13}C NMR), and AV II-600 (600.1 MHz for ^{1}H NMR; 150.9 MHz for ^{13}C NMR) instruments. All spectra were measured at 300 K, unless otherwise specified. The chemical shifts δ are given in ppm and referenced to the external standard TMS or internal solvent standard. The connectivity was determined by ^{1}H,^{1}H-COSY, ^{1}H,^{13}C-HSQC and ^{1}H,^{13}C-HMBC experiments. ^{1}H NMR coupling constants (*J*) are reported in Hertz (Hz), and multiplicities are indicated as follows: s (singlet), d (doublet), t (triplet), q (quartet), m (multiplet), dd (doublet of doublets). Mass spectra: Electrospray ionization (ESI) mass spectra were measured with a ThermoFisher Scientific LTQOrbitrap Velos (linear ion trap coupled with orbitrap mass analyser; resolution: 100000 FWHM at *m*/*z* = 400 u)

General experimental procedure for the preparation of imines [1] (1d,e)

A mixture of aldehyde (10 mmol), *p*-toluenesulfonamide (10 mmol) and sodium *p*-toluenesulfinate (10 mmol) in formic acid (15 mL) and H_2O (15 mL) was stirred for 16–24 h at rt. The resulting white precipitate was filtered off, washed with H_2O (3 × 10 mL), then pentane (2 × 10 mL), and dissolved in CH_2Cl_2 (100 mL). Sat. aq $NaHCO_3$ (70 mL) was added and the solution was stirred for 2 h at rt. The organic phase was collected, the aqueous phase extracted with CH_2Cl_2 (3 × 70 mL) and the combined organic layers dried ($MgSO_4$), filtered and the solvent removed under high vacuum to yield the corresponding sulfonylimine. Aliphatic imines were used without further purification, and aromatic imines were purified by precipitation from CH_2Cl_2 with hexane.

General experimental procedure for the preparation of 4-substituted-3(2*H*)-furanones (3, 5–14):

Imine (1.0 equiv), $Pd_2dba_3{\cdot}CHCl_3$ (5 mol %), dppe (10 mol %), Na_2CO_3 (2.0 equiv) were placed in a Schlenk tube. The reaction setup was degassed and flushed with argon 3 times. To the mixture under argon was added dioxane (2 mL) followed by 4-chloroacetoacetate (1.1 equiv), whereupon the mixture was stirred at 50 °C for 10 h. After completion of the reaction, the mixture was diluted with

dichloromethane (20 mL) and washed with water (2 × 20 mL) and saturated brine (20 mL). The organic layer was then dried over anhydrous magnesium sulfate and the solvent was evaporated in vacuo. The residue was subjected to flash silica gel column chromatography using mixtures of ethyl acetate in pentane, affording the product in good to excellent yields.

Compound 3: Yield: 88% (131 mg), colourless viscous liquid, R_f : 0.76 (pentane/ethyl acetate = 3:7). - IR ν_{max} : 3276, 3062, 2993, 2962, 1687, 1577, 1484, 1450, 1423, 1388, 1328, 1261, 1163, 1082, 1009, 856, 799, 731, 699 cm^{-1}. - ^1H NMR (400 MHz, $CDCl_3$): δ = 7.65-7.63 (m, 2 H), 7.31-7.16 (m, 7 H), 6.43-6.40 (d, 1 H, *J* = 10 Hz), 5.24-5.22 (d, 1 H, *J* = 10 Hz), 4.36-4.31 (m, 3 H), 4.08-4.04 (d, 1 H, *J* = 16 Hz), 2.38 (s, 3 H), 1.40 ppm (t, 3 H, *J* = 7.2 Hz) - ^{13}C NMR (100 MHz, $CDCl_3$): δ = 194.3, 179.7, 142.9, 139.9, 137.8, 128.9, 128.5, 127.5, 127.2, 126.5, 93.4, 74.6, 66.7, 51.4, 21.4, 14.7 ppm. - HRMS: Calcd for $C_{20}H_{21}NO_5S$, $(M+Na)^+$: 410.1037; found: 410.1034.

Compound 5: Yield: 90% (128 mg), colourless viscous liquid, R_f : 0.78 (pentane/ethyl acetate = 3:7). - IR ν_{max} : 3260, 3061, 3030, 2952, 1691, 1581, 1484, 1452, 1422, 1388, 1329, 1158, 1126, 1084, 1040, 847, 783, 727, 697 cm^{-1}. - ^1H NMR (400 MHz, $CDCl_3$): δ = 7.65-7.62 (m, 2 H), 7.30-7.17 (m, 7 H), 6.39-6.37 (d, 1 H, *J* = 10 Hz), 5.23-5.21 (d, 1 H, *J* = 10 Hz), 4.35-4.31 (d, 1 H, *J* = 16 Hz), 4.10-4.06 (d, 1 H, *J* = 16 Hz), 3.96 (s, 3 H), 2.38 ppm (s, 3 H) - ^{13}C NMR (100 MHz, $CDCl_3$): δ = 194.3, 179.8, 142.9, 139.8, 137.7, 128.9, 128.5, 127.5, 127.1, 126.5, 93.5, 74.7, 56.6, 51.3, 21.4 ppm. - HRMS: Calcd for $C_{19}H_{19}NO_5S$, $(M+Na)^+$: 396.0881; found: 396.0876.

Compound 6:Yield: 93% (133 mg), colorless solid, m. p. : 135-137 ^oC. - IR ν_{max} : 3172, 3062, 2992, 2930, 1689, 1585, 1489, 1437, 1422, 1388, 1330, 1161, 1090, 1012, 906, 851, 727, 690 cm^{-1}. - ^1H NMR (400 MHz, $CDCl_3$): δ = 7.62-7.60 (m, 2 H), 7.24-7.16 (m, 6 H), 6.53-6.51 (d, 1 H, *J* = 9.6 Hz), 5.19-5.16 (d, 1 H, *J* = 9.6 Hz), 4.36-4.30 (m, 3 H), 4.12-4.08 (d, 1 H, *J* = 16 Hz), 2.39 (s, 3 H) 1.38 ppm (t, 3 H, *J* = 7.2 Hz)- ^{13}C NMR (100 MHz, $CDCl_3$): δ = 194.2, 179.6, 143.1, 138.5, 137.5, 133.2, 128.9, 128.4, 127.9, 126.9, 92.8, 74.6, 66.9, 50.8, 21.3, 14.6 ppm. - HRMS: Calcd for $C_{20}H_{20}ClNO_5S$, $(M+Na)^+$: 444.0648; found: 444.0644.

Compound 7:Yield: 96% (133 mg), colorless solid, m. p. : 146-148 ^oC. - IR ν_{max} : 3092, 3062, 2953, 2927, 1678, 1568, 1493, 1455, 1395, 1327, 1160, 1131, 1091, 1060, 1012, 961, 862, 818, 767, 694 cm^{-1}. - ^1H NMR (400 MHz, $CDCl_3$): δ = ^1H NMR (400 MHz, $CDCl_3$): δ = 7.62-7.60 (m, 2 H), 7.22-7.16 (m, 6 H), 6.37-6.34 (d, 1 H, *J* = 9.6 Hz), 5.20-5.18 (d, 1 H, *J* = 9.6 Hz), 4.39-4.35 (d, 1 H, *J* = 16 Hz), 4.16-4.11 (d, 1 H, *J* = 16 Hz), 3.99 (s, 3 H), 2.39 ppm (s, 3 H) - ^{13}C NMR (100 MHz, $CDCl_3$): δ = 194.2, 179.9, 143.1, 138.4, 137.7, 133.4, 129.1, 128.6, 128.0, 127.2, 93.3, 74.9, 56.8, 50.9, 21.5 ppm. - HRMS: Calcd for $C_{19}H_{18}ClNO_5S$, $(M+Na)^+$: 430.0491; found: 430.0488.

Compound 8: Yield: 71% (102 mg), colourless viscous liquid, R_f : 0.78 (pentane/ethyl acetate = 3:7). - IR ν_{max} : 3264, 3062, 2992, 2939, 1687, 1585, 1511, 1485, 1443, 1388, 1334, 1304, 1247, 1159, 1093, 1031, 909, 855, 816, 777, 724, 663 cm^{-1}. - ^1H NMR (400 MHz, $CDCl_3$): δ = 7.64-7.62

(m, 2 H), 7.22-7.17 (m, 4 H), 6.76-6.74 (m, 2 H), 6.39-6.37 (d, 1 H, *J* = 9.6 Hz), 5.19-5.16 (d, 1 H, *J* = 9.6 Hz), 4.35-4.29 (m, 3 H), 4.06-4.02 (d, 1 H, *J* = 16 Hz), 3.75 (s, 3 H), 2.38 (s, 3 H), 1.39 ppm (t, 3 H, *J* = 7.2 Hz) - ^{13}C NMR (100 MHz, $CDCl_3$): δ = 194.3, 179.6, 158.9, 142.8, 137.8, 132.1, 128.9, 127.8, 127.1, 113.7, 93.5, 74.6, 66.7, 55.2, 50.9, 21.4, 14.7 ppm. - HRMS: Calcd for $C_{21}H_{23}NO_6S$, $(M+Na)^+$: 440.1143; found: 440.1139.

Compound 9: Yield: 78% (109 mg), colorless solid, m. p. : 120-122 ^{o}C. - IR ν_{max} : 3085, 3008, 2957, 2929, 1678, 1566, 1509, 1491, 1455, 1390, 1320, 1248, 1158, 1128, 1094, 1058, 1032, 955, 930, 862, 786, 766, 727, 659 cm^{-1}. - ^{1}H NMR (400 MHz, $CDCl_3$): δ = 7.64-7.62 (m, 2 H), 7.21-7.17 (m, 4 H), 6.76-6.74 (m, 2 H), 6.30-6.27 (d, 1 H, *J* = 10 Hz), 5.19-5.16 (d, 1 H, *J* = 9.6 Hz), 4.36-4.32 (d, 1 H, *J* = 16 Hz), 4.09-4.05 (d, 1 H, *J* = 16 Hz), 3.96 (s, 3 H), 3.75 (s, 3 H), 2.39 ppm (s, 3 H) - ^{13}C NMR (100 MHz, $CDCl_3$): δ = 194.3, 179.8, 159.0, 142.9, 137.9, 132.1, 128.9, 127.8, 127.2, 113.8, 93.7, 74.7, 56.6, 55.2, 50.9, 21.4 ppm. - HRMS: Calcd for $C_{20}H_{21}NO_6S$, $(M+Na)^+$: 426.0987; found: 426.0984.

Compound 10: Yield: 78% (119 mg), colorless solid, m. p. : 67-69 ^{o}C. - IR ν_{max} : 3266, 2957, 2932, 1688, 1586, 1485, 1443, 1388, 1336, 1160, 1092, 1034, 1006, 909, 856, 815, 726, 664 cm^{-1}. - ^{1}H NMR (400 MHz, $CDCl_3$): δ = 7.67-7.64 (m, 2 H), 7.22-7.20 (d, 2 H, *J* = 8 Hz), 5.78-5.76 (d, 1 H, *J* = 10.4 Hz), 4.34-4.22 (m, 3 H), 4.02-3.96 (m, 1 H), 3.89-3.86 (d, 1 H, *J* = 16 Hz), 2.39 (s, 3 H), 1.72-1.65 (m, 1 H), 1.58-1.51 (m, 1 H), 1.40 (t, 3 H, *J* = 6.8 Hz), 1.29-1.25 (m, 4 H), 0.85 ppm (t, 3 H, *J* = 7.2 Hz) - ^{13}C NMR (100 MHz, $CDCl_3$): δ = 194.6, 179.6, 142.8, 137.9, 128.9, 127.0, 93.1, 74.3, 66.3, 47.9, 34.9, 27.7, 21.9, 21.4, 14.7, 13.8 ppm. - HRMS: Calcd for $C_{18}H_{25}NO_5S$, $(M+Na)^+$: 390.1350; found: 390.1347.

Compound 11:Yield: 80% (130 mg), colorless solid, m. p. : 111-113 ^{o}C. - IR ν_{max} : 3257, 2950, 1695, 1596, 1480, 1442, 1393, 1350, 1328, 1302, 1157, 1106, 1092, 1064, 1036, 1006, 907, 818, 729, 664 cm^{-1}. - ^{1}H NMR (400 MHz, $CDCl_3$): δ = 7.67-7.65 (m, 2 H), 7.23-7.21 (d, 2 H, *J* = 8 Hz), 5.65-5.63 (d, 1 H, *J* = 10.4 Hz), 4.27-4.23 (d, 1 H, *J* = 16 Hz), 4.02-3.96 (m, 1 H), 3.93 (s, 3 H), 3.91-3.87 (d, 1 H, *J* = 15.6 Hz), 2.40 (s, 3 H), 1.74-1.65 (m, 1 H), 1.59-1.49 (m, 1 H), 1.29-1.24 (m, 4 H), 0.84 ppm (t, 3 H, *J* = 7.2 Hz) - ^{13}C NMR (100 MHz, $CDCl_3$): δ = 194.6, 179.8, 142.8, 138.1, 128.9, 127.2, 93.4, 74.5, 56.4, 47.9, 34.9, 27.8, 22.1, 21.4, 13.9 ppm. - HRMS: Calcd for $C_{17}H_{23}NO_5S$, $(M+Na)^+$: 376.1194; found: 376.1190.

Compound 12: Yield: 72% (104 mg), colorless solid, m. p. : 141-143 ^{o}C. - IR ν_{max} : 3139, 3067, 3030, 2975, 2926, 1678, 1576, 1490, 1438, 1383, 1355, 1333, 1180, 1152, 1126, 1095, 1038, 1012, 980, 964, 850, 813, 733, 656 cm^{-1}. - ^{1}H NMR (600 MHz, $CDCl_3$): δ = 7.67-7.66 (d, 2 H, *J* = 7.8 Hz), 7.25-7.11 (m, 7 H), 5.80-5.78 (d, 1 H, *J* = 10.2 Hz), 4.32-4.23 (m, 2 H), 4.21-4.18 (d, 1 H, *J* = 15.6 Hz), 4.09-4.05 (m, 1 H), 3.88-3.86 (d, 1 H, *J* = 16.2 Hz), 2.74-2.68 (m, 1 H), 2.59-2.54 (m, 1 H), 2.39 (s, 3 H), 2.06-2.00 (m, 1 H), 1.92-1.86 (m, 1 H), 1.39 ppm (t, 3 H, *J* = 7.2 Hz) - ^{13}C NMR

(150 MHz, $CDCl_3$): δ = 194.6, 179.5, 142.9, 141.2, 137.9, 128.9, 128.3, 128.2, 127.1, 125.8, 92.9, 74.4, 66.4, 47.9, 36.7, 32.1, 21.5, 14.7 ppm. - HRMS: Calcd for $C_{22}H_{25}NO_5S$, $(M+Na)^+$: 438.1350; found: 438.1348.

Compound 13:Yield: 78% (108 mg), colorless solid, m. p. : 143-145 ^oC. - IR ν_{max} : 3247, 3036, 3001, 2959, 1687, 1582, 1488, 1439, 1418, 1393, 1360, 1325, 1158, 1090, 1047, 1000, 974, 936, 895, 815, 732, 667 cm^{-1}. - ^1H NMR (600 MHz, $CDCl_3$): δ = 7.67-7.66 (d, 2 H, *J* = 8.4 Hz), 7.25-7.11 (m, 7 H), 5.82-5.79 (d, 1 H, *J* = 10.2 Hz), 4.21-4.18 (d, 1 H, *J* = 16.2 Hz), 4.07-4.03 (m, 1 H), 3.91-3.88 (m, 4 H), 2.73-2.68 (m, 1 H), 2.58-2.53 (m, 1 H), 2.39 (s, 3 H), 2.05-2.00 (m, 1 H), 1.92-1.87 ppm (m, 1 H) - ^{13}C NMR (150 MHz, $CDCl_3$): δ = 194.6, 179.6, 142.9, 141.1, 137.8, 128.9, 128.3, 128.2, 127.1, 125.8, 93.0, 74.5, 56.4, 47.7, 36.5, 32.1, 21.4 ppm. - HRMS: Calcd for $C_{21}H_{23}NO_5S$, $(M+Na)^+$: 424.1194; found:. 424.1191.

Compound 14: Yield: 82% (150 mg), light yellow viscous liquid, R_f : 0.2 (pentane/ethyl acetate = 1:1). - IR ν_{max} : 3402, 2979, 2934, 1710, 1594, 1495, 1442, 1388, 1352, 1313, 1245, 1163, 1010, 856, 805, 727, 698 cm^{-1}. - ^1H NMR (600 MHz, $CDCl_3$): δ = 7.41-7.40 (m, 2H), 7.31-7.28 (m, 2H), 7.23- 7.20 (m, 1H), 6.56-6.55 (d, *J* = 8.3 Hz, 1H), 5.55-5.54 (d, *J* = 8.3 Hz, 1H), 4.57-4.50 (m, 2H), 4.47- 4.43 (m, 2H) , 1.43-1.42 (m, 12H). - ^{13}C NMR (150 MHz, $CDCl_3$): δ = 194.9, 180.1, 155.2, 142.7, 128.4, 127.1, 126.2, 94.8, 79.3, 74.8, 66.6, 48.4, 28.4, 14.7 ppm. - HRMS: Calcd for $C_{18}H_{23}NO_5$, $(M)^+$: 333.1576; found:. 333.1571.

General experimental procedure for the preparation of 4-hydrazino-3(2*H*)-furanones (20–25):
$Pd(PPh_3)_4$ (5 mol %) and Na_2CO_3 (2.0 equiv) were placed in a Schlenk tube. The reaction setup was degassed and flushed with argon 3 times. To the mixture under argon was added dioxane (2 mL) followed by 4-chloroacetoacetate (1.1 equiv) and diazo ester (1.0 equiv), whereupon the mixture was stirred at 50 °C for 10 h. After completion of the reaction, the mixture was diluted with dichloromethane (20 mL) and washed with water (2 × 20 mL) and saturated brine (20 mL). The organic layer was then dried over anhydrous magnesium sulfate and the solvent was evaporated in vacuo. The residue was subjected to flash silica gel column chromatography using mixtures of ethyl acetate in dichloromethane, affording the product in good to excellent yields.

Compound 20:Yield: 90% (180 mg), light yellow viscous liquid, R_f : 0.77 (dichloromethane/ethyl acetate = 2:8). - IR ν_{max} : 3272, 2939, 1716, 1593, 1451, 1373, 1354, 1231, 1179, 1145, 1106, 1066, 1036 cm^{-1}. - ^1H NMR (600 MHz, $CDCl_3$): δ = 7.08 (brs, 1H), 4.96-4.95 (m, 2 H), 4.65 (brs, 2 H), 4.62 (s, 2 H), 1.47 (t, 3 H, *J* = 7.2 Hz), 1.26 (s, 6H), 1.25 ppm (s, 6 H) - ^{13}C NMR (150 MHz, $CDCl_3$, Rotamer signals were seen): δ = 191.3, 190.5, 178.9, 178.6, 155.5, 155.3, 154.7, 154.5, 100.1, 73.9, 71.1, 69.7, 67.7, 22.0, 21.9, 14.8 ppm. - HRMS: Calcd for $C_{14}H_{22}N_2O_7$, $(M+Na)^+$: 353.1324; found: 353. 1319.

Compound 21: Yield: 94% (212 mg), light yellow viscous liquid, R_f : 0.79 (dichloromethane/ethyl

acetate = 2:8). - IR ν_{max} : 3270, 2940, 1714, 1596, 1490, 1396, 1372, 1314, 1231, 1179, 1145, 1107, 1071, 1037 cm^{-1}. - ^1H NMR (600 MHz, $CDCl_3$): δ = 7.08 (brs, 1H), 4.95-4.94 (m, 2 H), 4.63 (s, 2 H), 4.25 (brs, 3 H), 1.26 (s, 6H), 1.25 ppm (s, 6 H) - ^{13}C NMR (150 MHz, $CDCl_3$, Rotamer signals were seen): δ = 191.4, 190.5, 179.3, 178.9, 155.3, 155.2, 154.7, 154.4, 100.0, 99.9, 74.0, 71.2, 69.8, 57.7, 57.6, 22.0, 21.9 ppm. - HRMS: Calcd for $C_{13}H_{20}N_2O_7$, $(M+Na)^+$: 339.1168; found: 339. 1163.

Compound 22: Yield: 90% (155 mg), light yellow viscous liquid, R_f : 0.82 (dichloromethane/ethyl acetate = 2:8). - IR ν_{max} : 3269, 2939, 1721, 1591, 1449, 1375, 1330, 1225, 1183, 1094, 1060, 1035 cm^{-1}. - ^1H NMR (600 MHz, $CDCl_3$): δ = 7.2 (brs, 1H), 4.62 (brs, 4 H), 4.22-4.17 (m, 4 H), 1.47 (t, 3 H, *J* = 7.2 Hz), 1.27 ppm (t, 6 H, *J* = 7.2 Hz) - ^{13}C NMR (150 MHz, $CDCl_3$, Rotamer signals were seen): δ = 190.4, 190.3, 178.9, 178.6, 155.8, 155.2, 100.0, 73.9, 67.7, 67.6, 63.3, 61.9, 14.8, 14.4 ppm. - HRMS: Calcd for $C_{12}H_{18}N_2O_7$, $(M+Na)^+$: 325.1011; found: 325. 1007.

Compound 23: Yield: 92% (151 mg), light yellow viscous liquid, R_f : 0.83 (dichloromethane/ethyl acetate = 2:8). - IR ν_{max} : 3273, 2941, 1719, 1592, 1491, 1394, 1374, 1327, 1225, 1182, 1068, 1029 cm^{-1}. - ^1H NMR (600 MHz, $CDCl_3$): δ = 7.2 (brs, 1H), 4.64 (s, 2 H), 4.25-4.17 (m, 7 H), 1.27 ppm (t, 6 H, *J* = 7.2 Hz) - ^{13}C NMR (150 MHz, $CDCl_3$, Rotamer signals were seen): δ = 191.3, 190.5, 179.3, 155.6, 155.0, 100.0, 74.1, 63.1, 62.0, 57.7, 57.6, 14.4 ppm. - HRMS: Calcd for $C_{11}H_{16}N_2O_7$, $(M+Na)^+$: 311.0855; found: 311. 0851.

Compound 24: Yield: 86% (203 mg), light yellow viscous liquid, R_f : 0.67 (dichloromethane/ethyl acetate = 2:8). - IR ν_{max} : 3270, 2935, 1714, 1597, 1485, 1451, 1388, 1349, 1242, 1150, 1067 cm^{-1}. - ^1H NMR (600 MHz, $CDCl_3$): δ = 6.9 (brs, 1H), 4.68 (brs, 2 H), 4.59 (s, 2 H), 1.46 ppm (s, 21 H) - ^{13}C NMR (150 MHz, $CDCl_3$, Rotamer signals were seen): δ = 191.7, 190.5, 178.9, 178.3, 154.4, 153.8, 100.2, 100.1, 82.3, 81.1, 81.0, 73.7, 67.7, 67.6, 28.2, 28.0, 14.9 ppm. - HRMS: Calcd for $C_{16}H_{26}N_2O_7$, $(M+Na)^+$: 381.1637; found: 381. 1633.

Compound 25: Yield: 86% (209 mg), light yellow viscous liquid, R_f : 0.71 (dichloromethane/ethyl acetate = 2:8). - IR ν_{max} : 3272, 2933, 1714, 1598, 1489, 1394, 1365, 1244, 1149, 1072, 1021 cm^{-1}. - ^1H NMR (600 MHz, $CDCl_3$): δ = 6.9 (brs, 1H), 4.61 (s, 2 H), 4.27 (s, 3 H), 1.46 ppm (s, 18 H) - ^{13}C NMR (150 MHz, $CDCl_3$, Rotamer signals were seen): δ = 191.8, 190.7, 179.3, 178.6, 154.6, 153.8, 100.8, 100.1, 82.4, 81.2, 73.9, 57.9, 57.8, 28.2, ppm. - HRMS: Calcd for $C_{15}H_{24}N_2O_7$, $(M+Na)^+$: 367.1481; found: 367. 1476.

Compound 27: Yield: 85% (200 mg), pale solid, m. p. : 101-103 ^oC. - IR ν_{max} : 3263, 2953, 2928, 1733, 1579, 1540, 1454, 1372, 1325, 1154, 1092, 1054, 990 cm^{-1}. ^1H NMR (400 MHz, $CDCl_3$): δ = 7.72-7.70 (d, 2 H, *J* = 7.6 Hz), 7.26-7.24 (d, 2 H, *J* = 8 Hz), 6.5 (m, 1 H), 6.19-6.17 (d, 1 H, *J* = 8.8 Hz), 4.25-4.22 (d, 1 H, *J* = 15.2 Hz), 4.08-4.02 (m, 1 H), 3.90-3.86 (d, 1 H, *J* = 15.2 Hz), 3.28-3.23 (m, 2 H), 2.42 (s, 3 H), 1.77-1.71 (m, 2 H), 1.59-1.57 (m, 2 H), 1.32-1.23 (m, 12 H), 0.90-0.88 (m, 3 H), 0.81 ppm (t, 3 H, *J* = 6 Hz) - ^{13}C NMR (75 MHz, $CDCl_3$): δ = 195.6, 176.5, 143.1, 137.8, 129.1,

126.8, 91.1, 73.7, 49.3, 41.6, 34.5, 31.7, 29.9, 28.9, 28.3, 26.7, 22.6, 22.4, 21.5, 14.1, 14.0 ppm. - LRMS: Calcd for $C_{23}H_{36}N_2O_4S$, $(M+H)^+$: 437.2; found: 437.2.

X-ray structure determinations

Crystals were mounted in inert oil on glass fibres and transferred to the cold gas stream of the diffractometer (**7**: Oxford Diffraction Xcalibur E using monochromated Mo Kα radiation; **9**: Oxford Diffraction Nova A using mirror-focussed Cu Kα radiation). Absorption corrections were based on multiscans. The structures were refined anisotropically on F^2 using the program SHELXL-97 [2]. NH hydrogens were refined freely, methyls as rigid groups, other H using a riding model. Compound **7** is a racemate and crystallizes only by chance in a Sohncke space group; the structure was refined as a racemic twin.

Crystallographic data have been deposited with the Cambridge Crystallographic Data Centre as supplementary publications no. CCDC-922077 (**7**), -922078 (**10**). Copies of the data can be obtained free of charge from http://www.ccdc.cam.ac.uk/data_request/cif.

Crystallographic data for compounds **7** and **10**.

Compound	**7**	**10**
Formula	$C_{19}H_{18}ClNO_5S$	$C_{18}H_{25}NO_5S$
M_r	407.85	367.45
Habit	colourless tablet	colourless tablet
Cryst. size (mm)	0.4 × 0.35 × 0.25	0.3 × 0.2 × 0.1
Crystal system	orthorhombic	orthorhombic
Space group	$P2_12_12_1$	*Aba*2
Tepmerature (°C)	–173	–173
Cell constants:		
a (Å)	7.3039(2)	21.2069(9)
b (Å)	13.1184(4)	11.2045(5)
c (Å)	19.3373(6)	15.8053(7)
V (Å^3)	1852.83	3737.8
Z	4	8
D_x (Mg m^{-3})	1.462	1.306
μ (mm^{-1})	0.35	1.8
Transmissions	0.96 – 1.00	0.83 – 1.00
F(000)	848	1568
$2\theta_{max}$	60	152
Refl. measured	60505	31390
Refl. indep.	5386	3869
R_{int}	0.043	0.038
Parameters	251	233
$wR(F^2$, all refl.)	0.071	0.067
$R(F$, >4s(F))	0.030	0.026

Flack parameter	0.49(4)	–0.024(12)
S	1.04	1.07
max. Δρ (e Å^{-3})	0.37	0.15

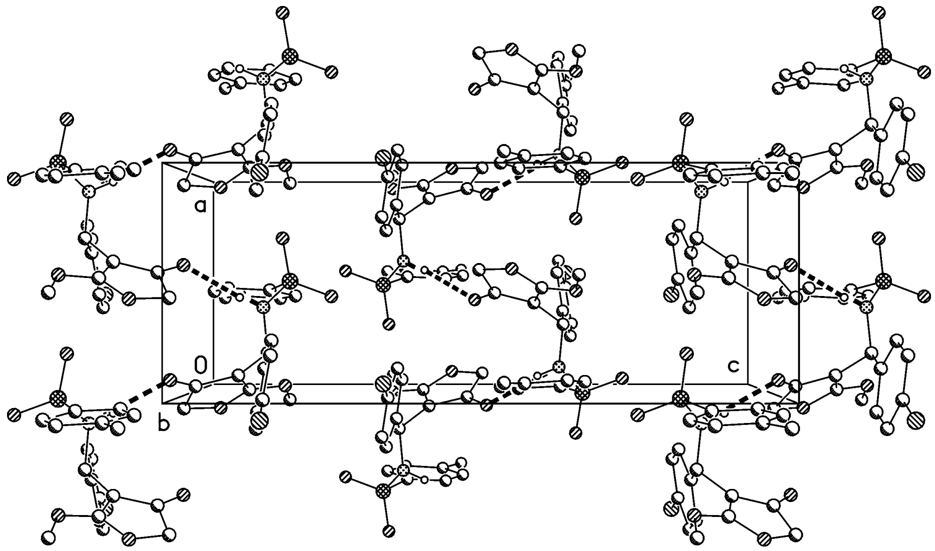

Packing diagram for compound **7**, showing chains of molecules linked by hydrogen bonds N1–H01···O2 (thick dashed lines) via the 2_1 axis parallel to *a*.

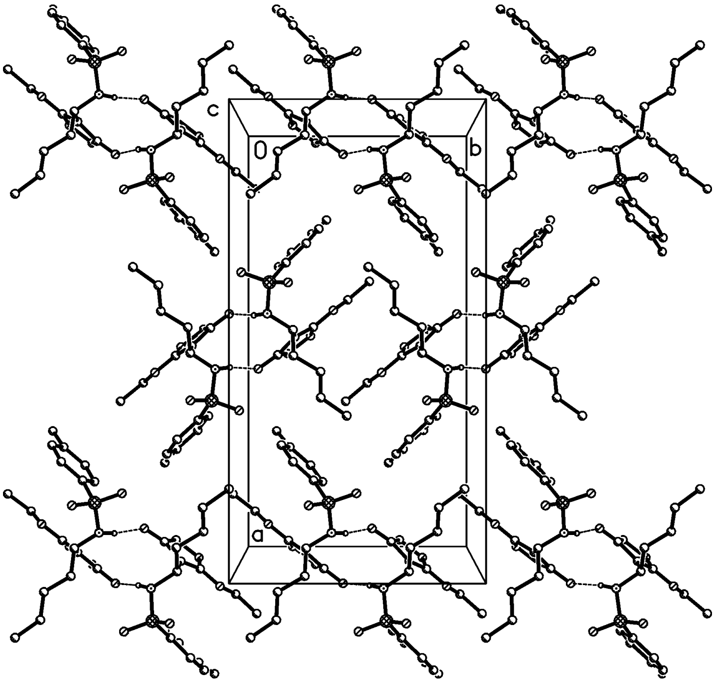

Packing diagram for compound **10**, showing pairs of molecules linked by hydrogen bonds N1–H01···O2 (thin dashed lines) via the twofold axis parallel to *a*.

References

1. F. Chemla, V. Hebbe, J.-F. Normant, *Synthesis* **2000**, 75–77.

2. G. M. Sheldrick, *Acta Cryst*. **2008**, *A64*, 112–122.

^{1}H-NMR (400 MHz)

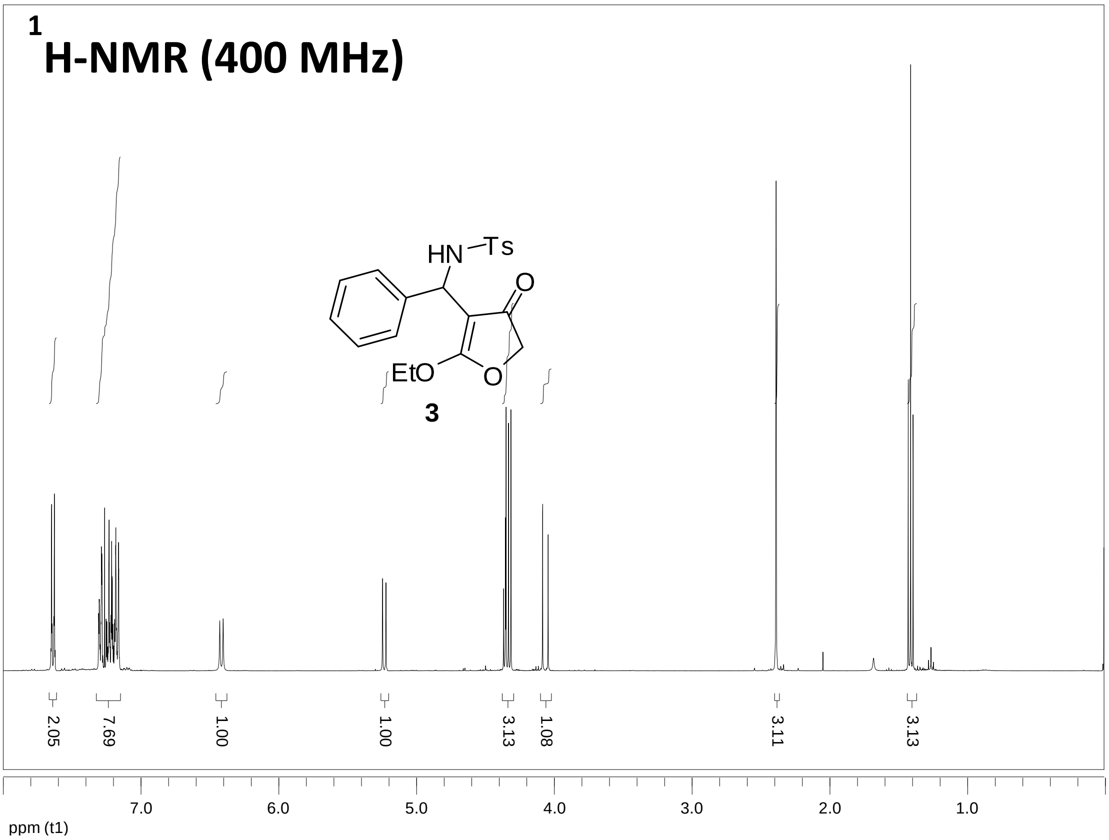

^{13}C-NMR (100 MHz)

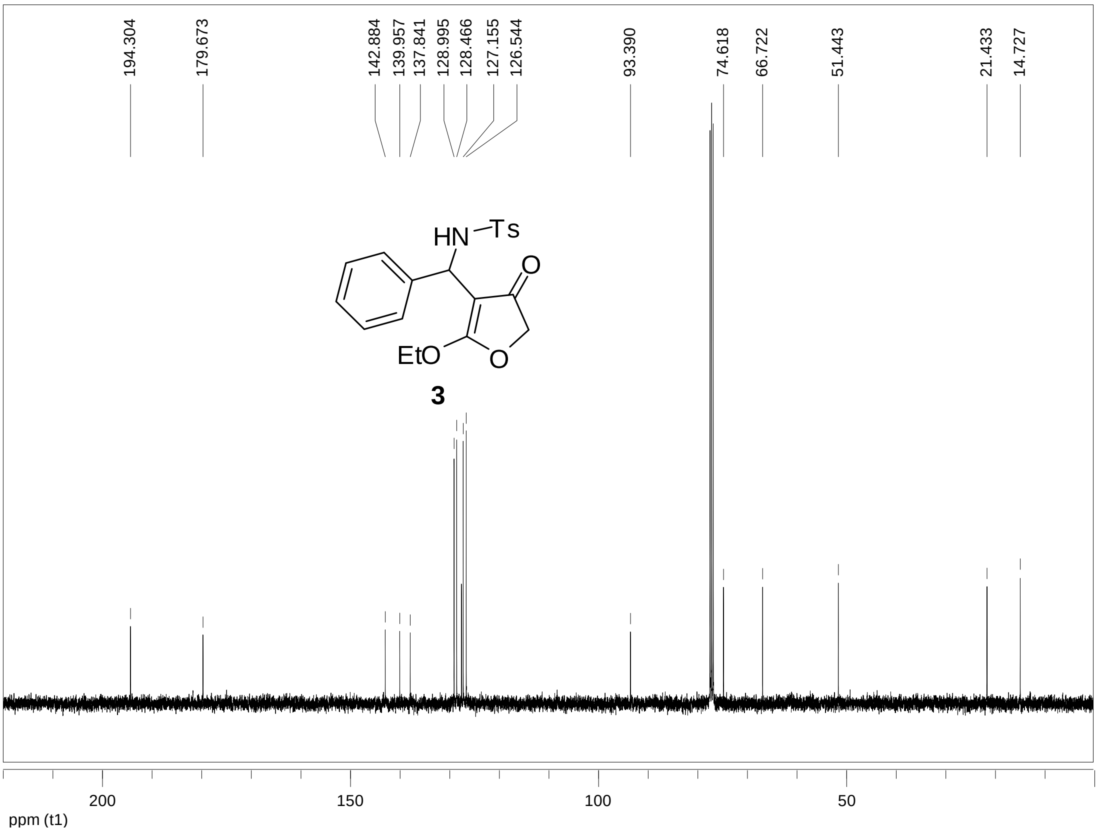

^{1}H-NMR (400 MHz)

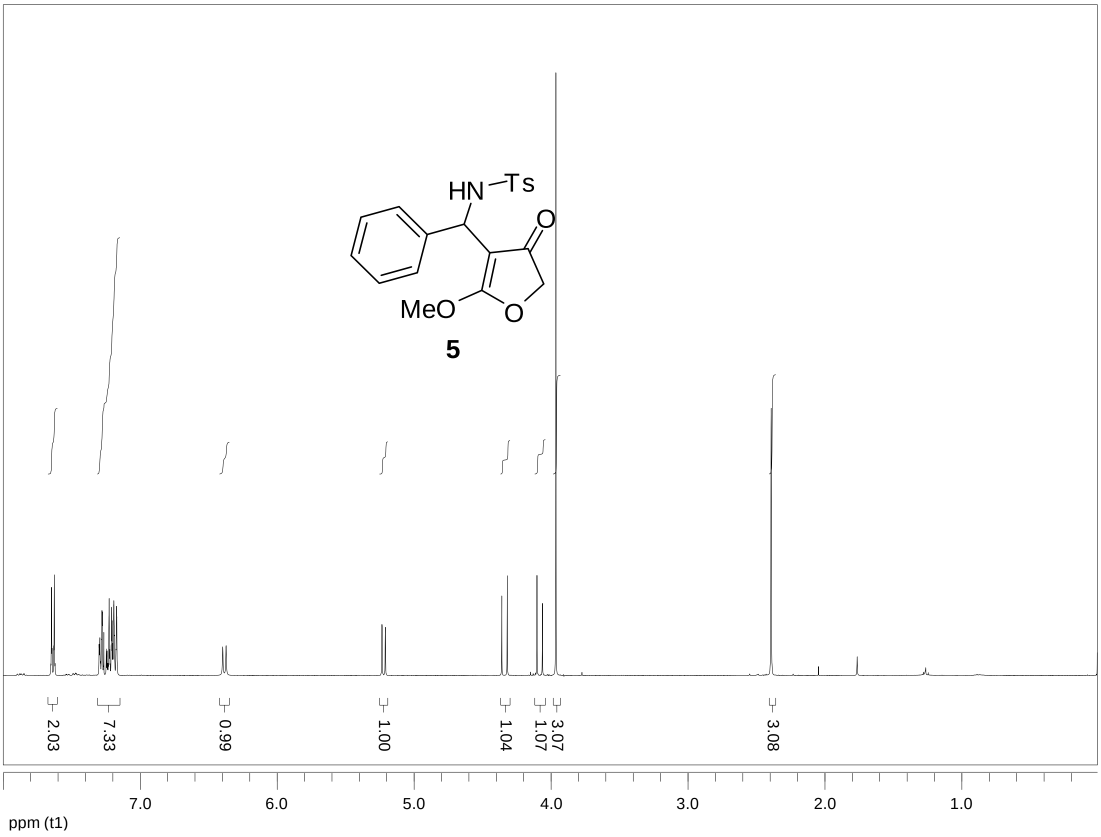

^{13}C-NMR (100 MHz)

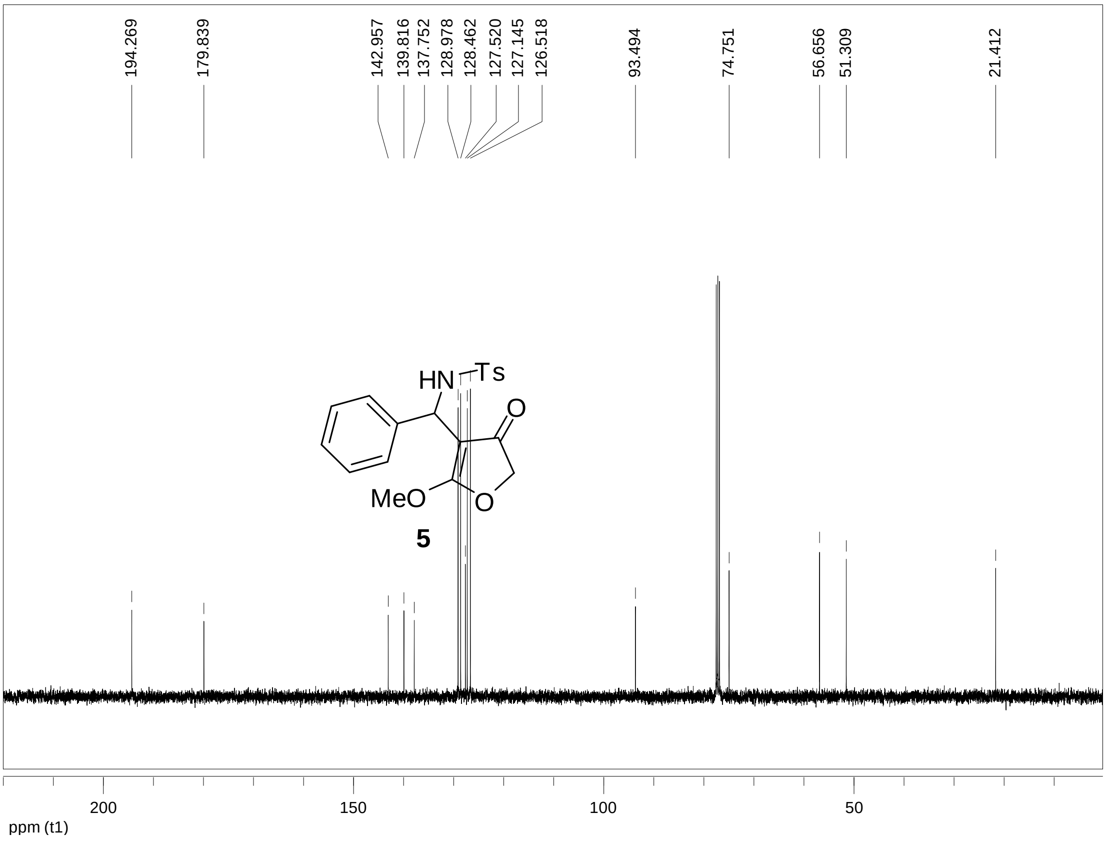

^{1}H-NMR (400 MHz)

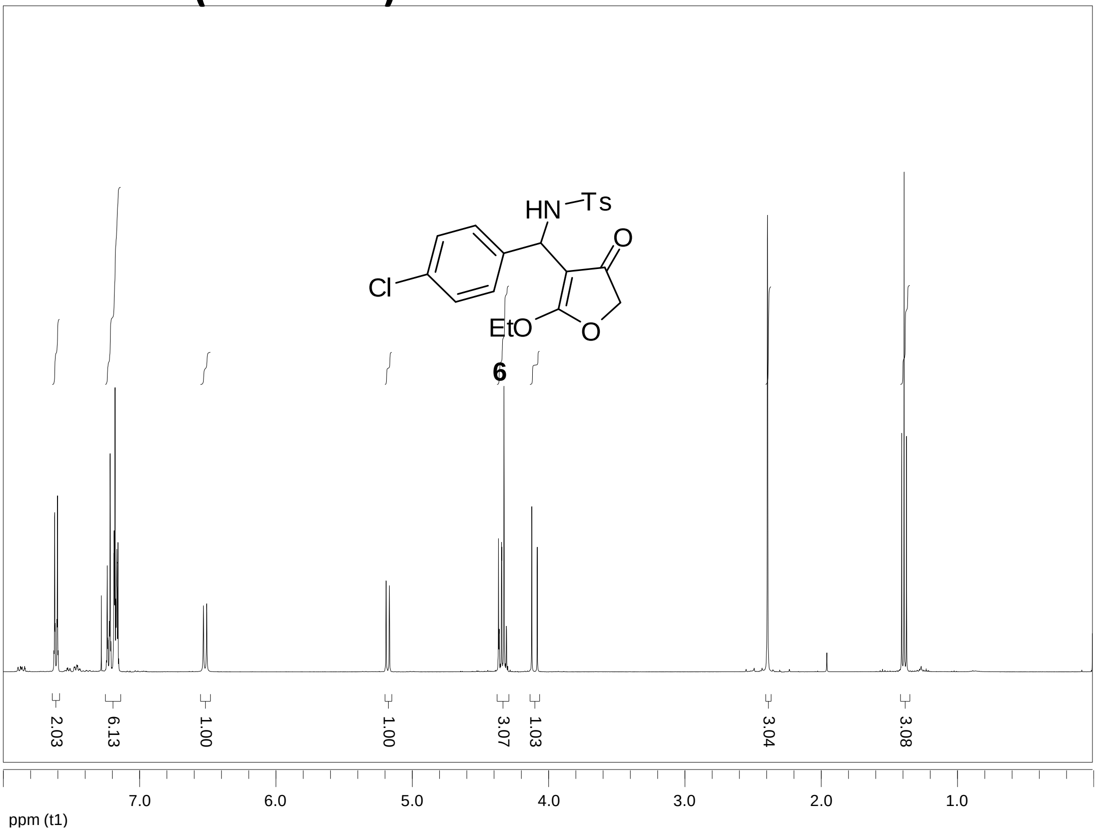

^{13}C-NMR (100 MHz)

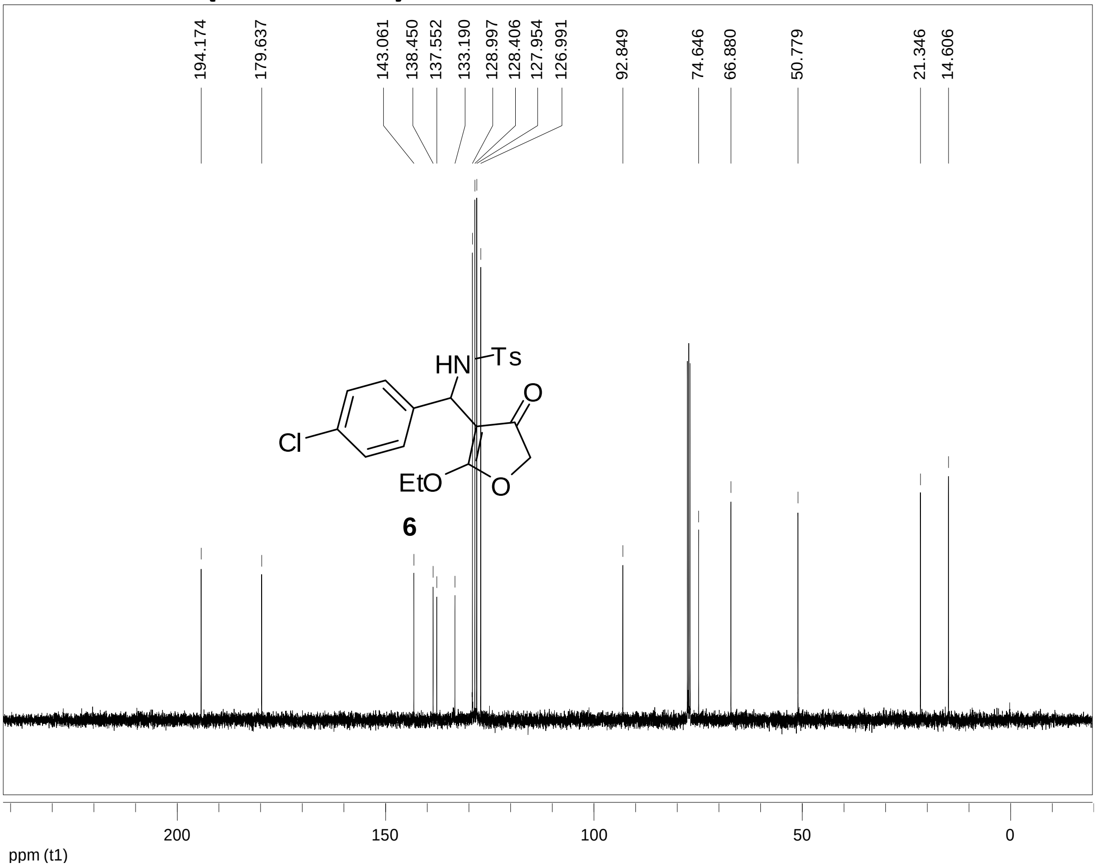

^{1}H-NMR (400 MHz)

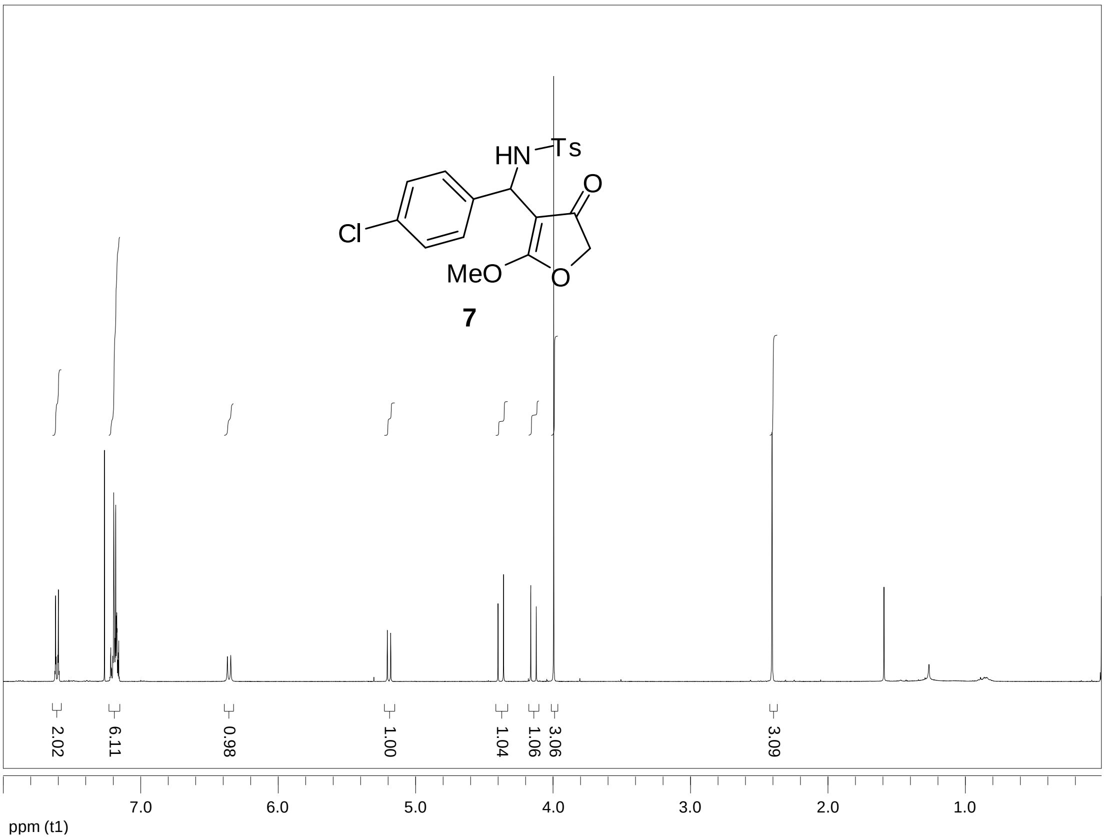

^{13}C-NMR (100 MHz)

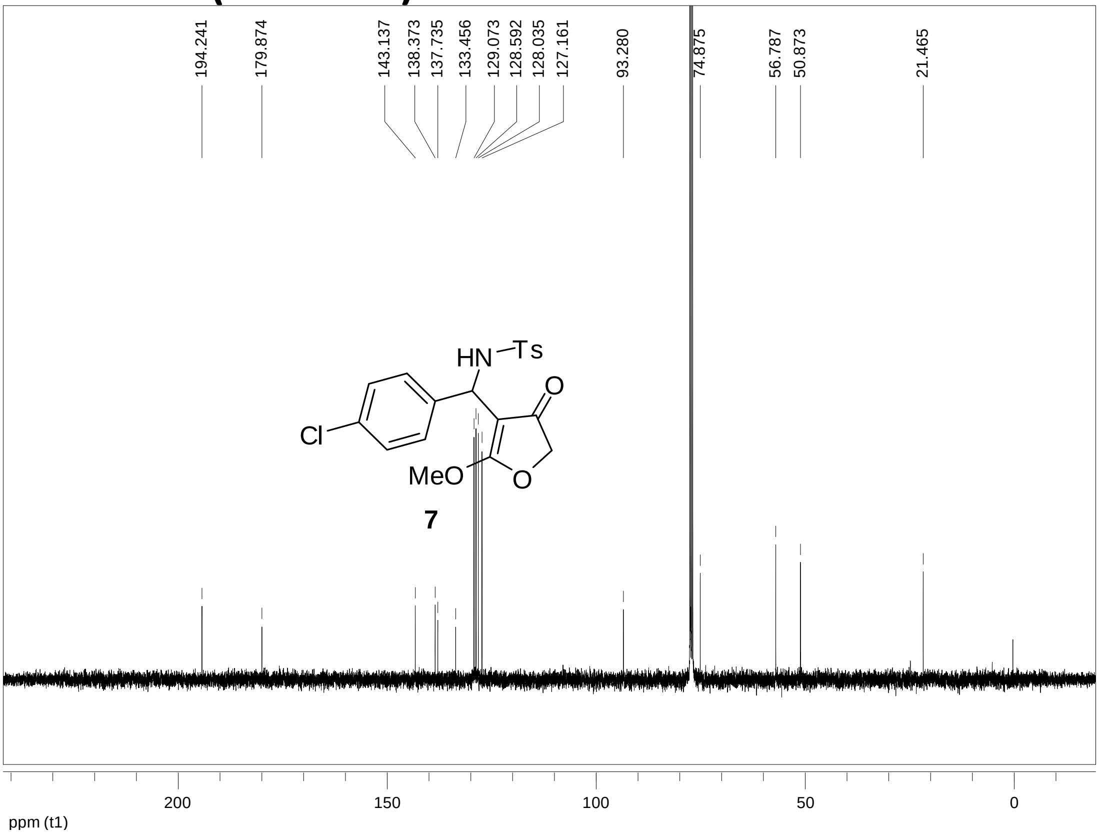

^{1}H-NMR (400 MHz)

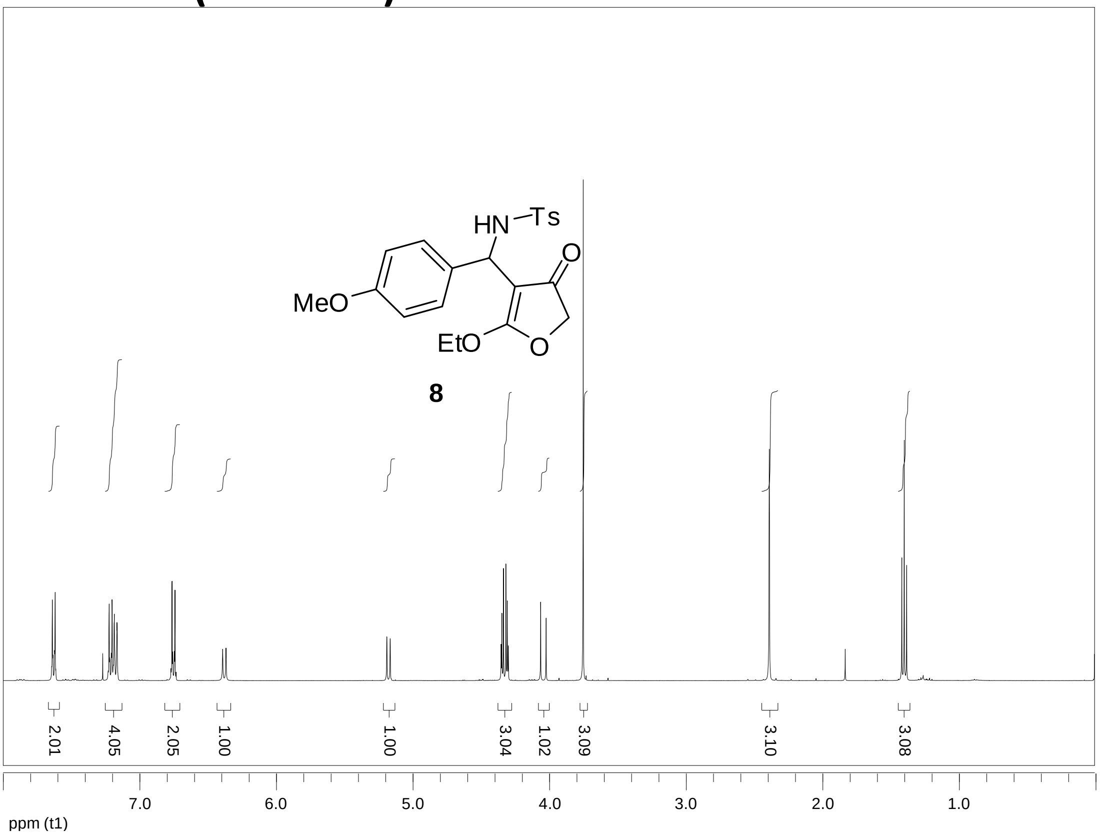

^{13}C-NMR (100 MHz)

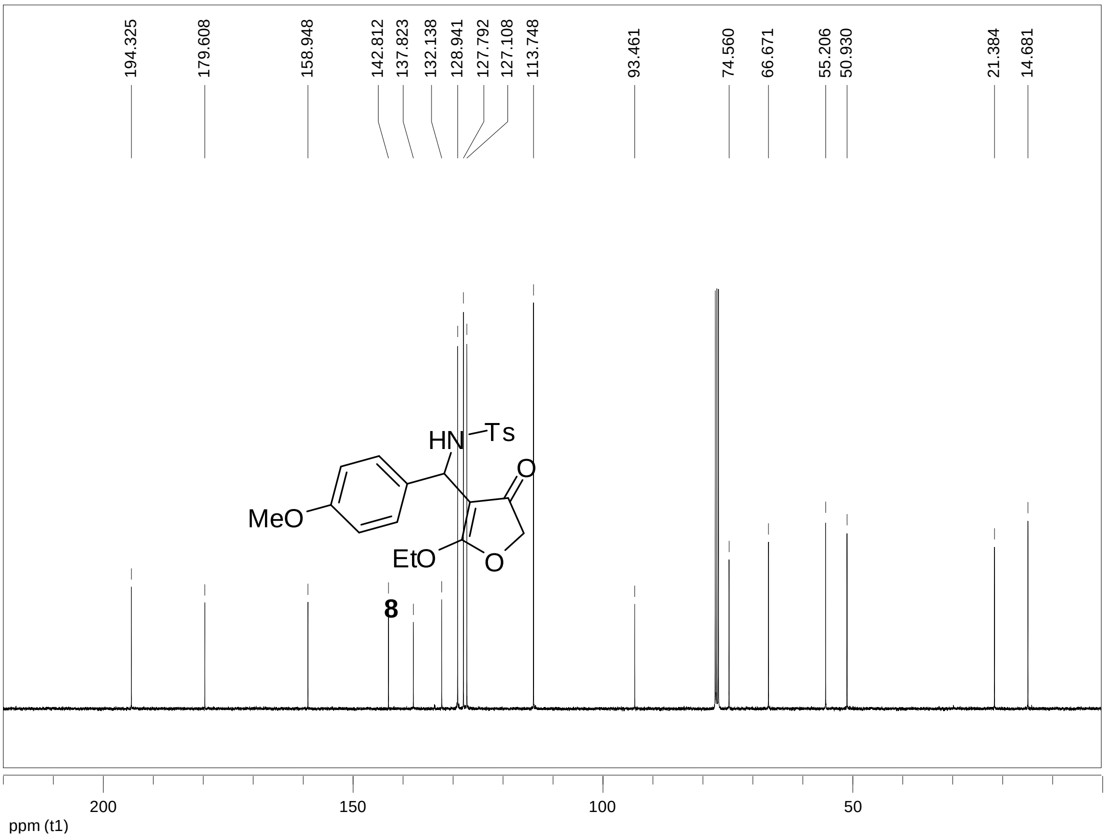

^{1}H-NMR (400 MHz)

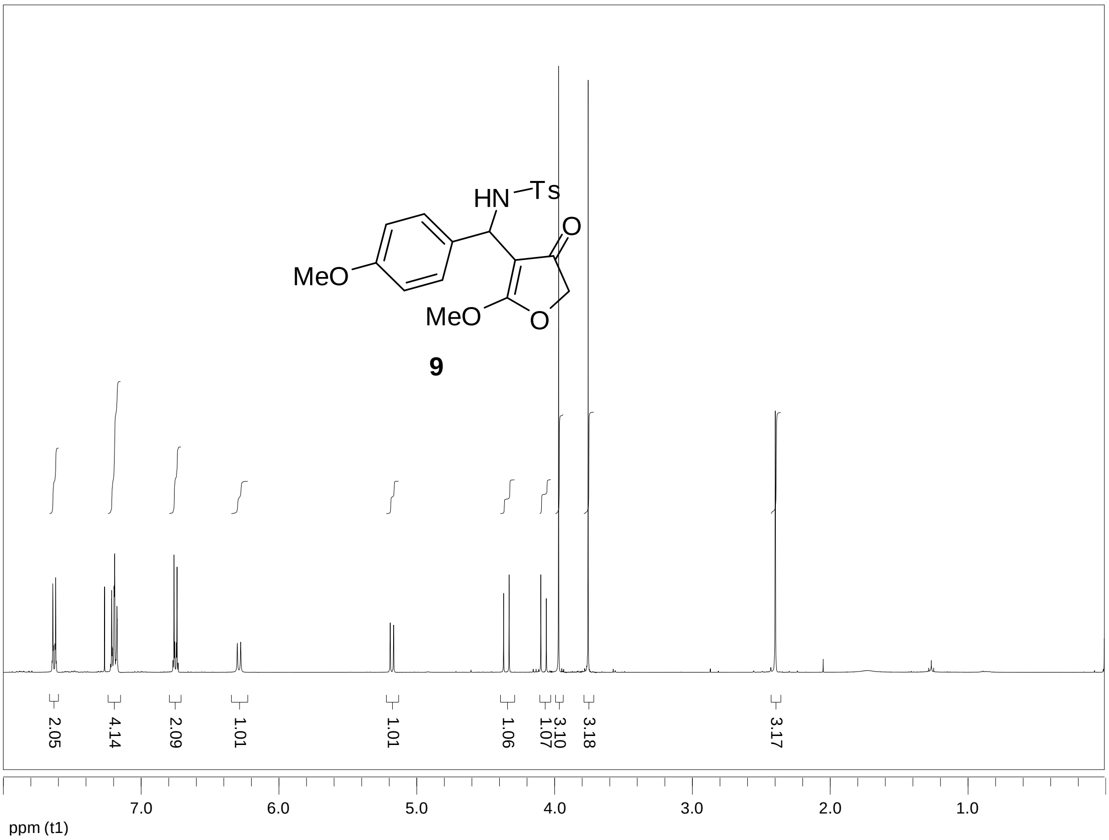

^{13}C-NMR (100 MHz)

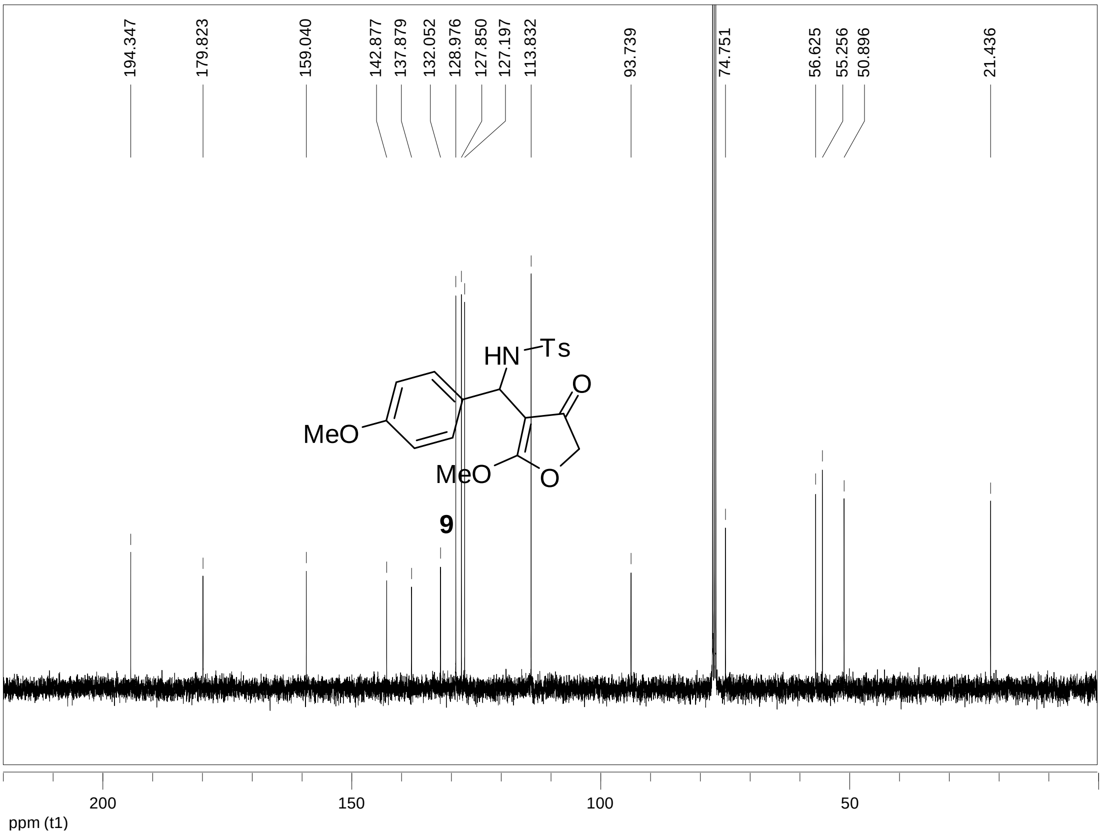

¹H-NMR (400 MHz)

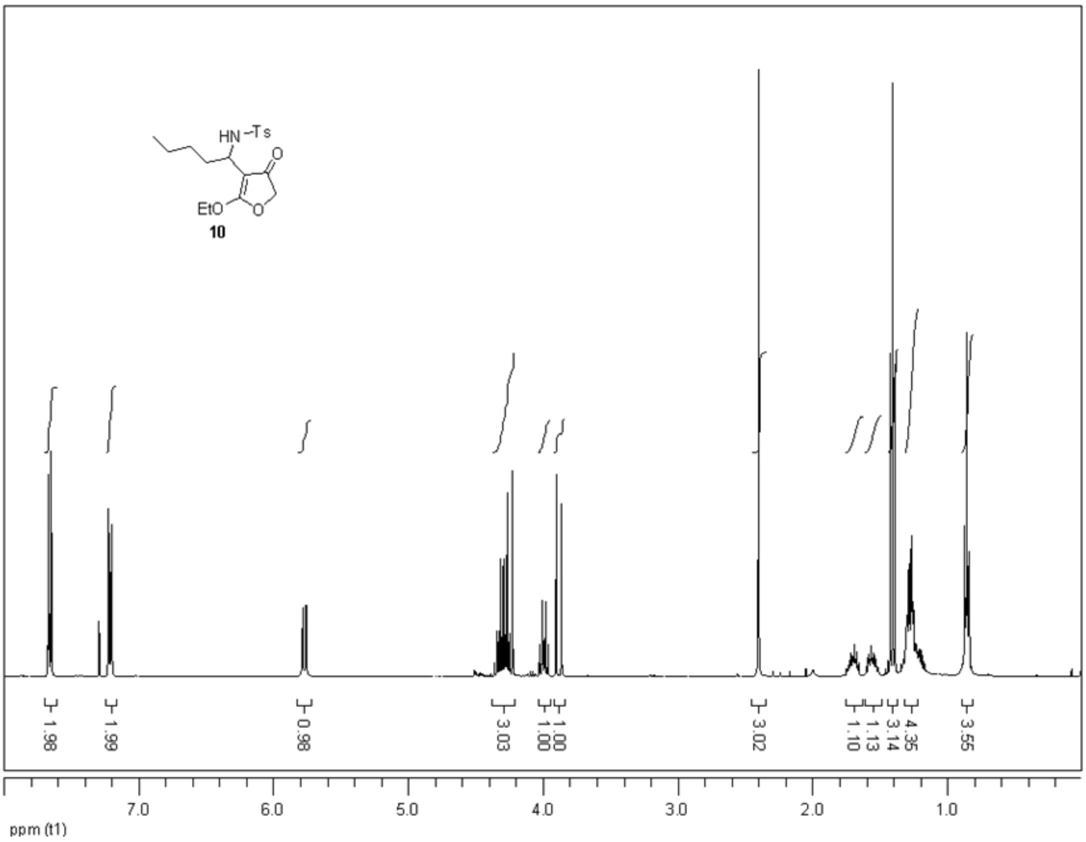

¹³C-NMR (100 MHz)

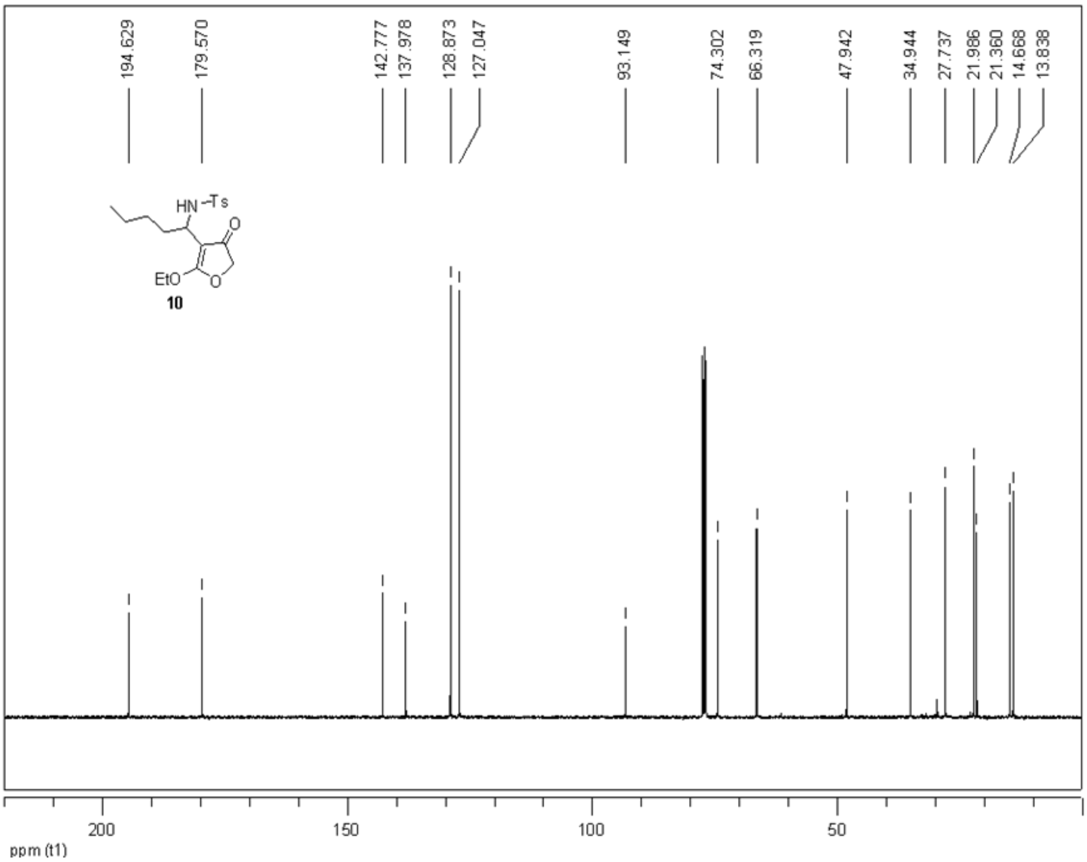

^{1}H-NMR (400 MHz)

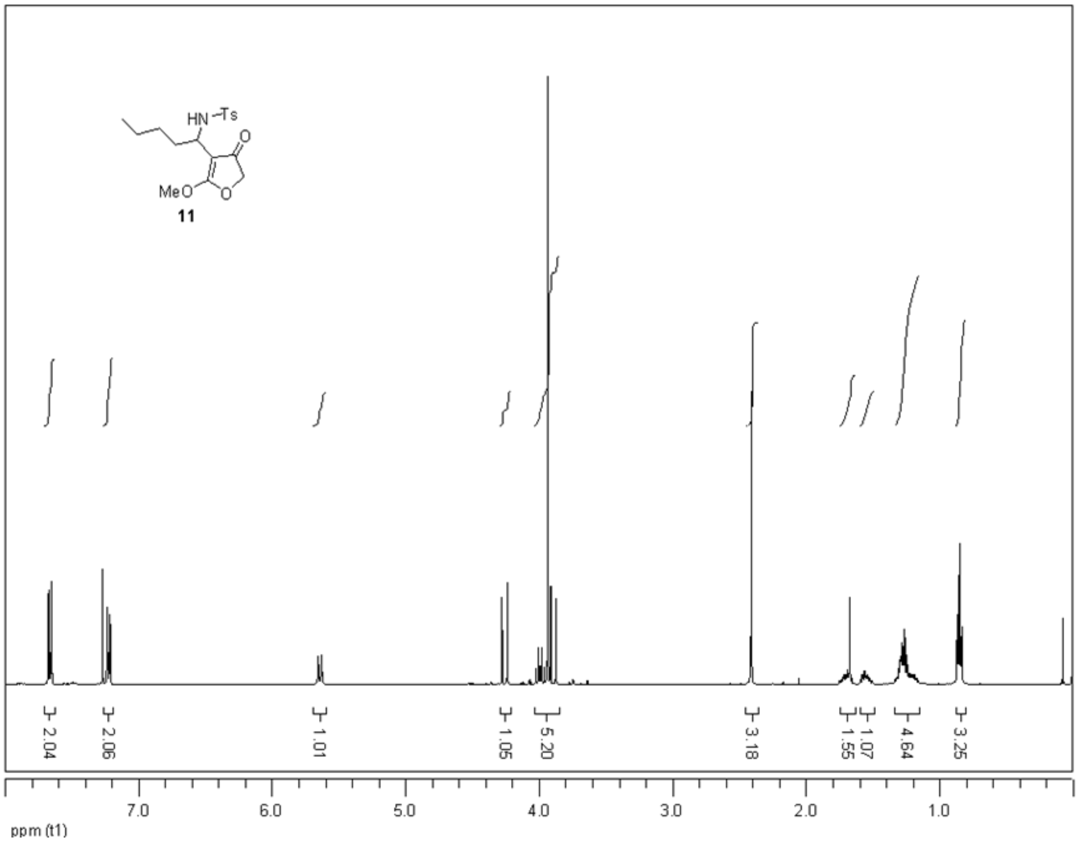

^{13}C-NMR (100 MHz)

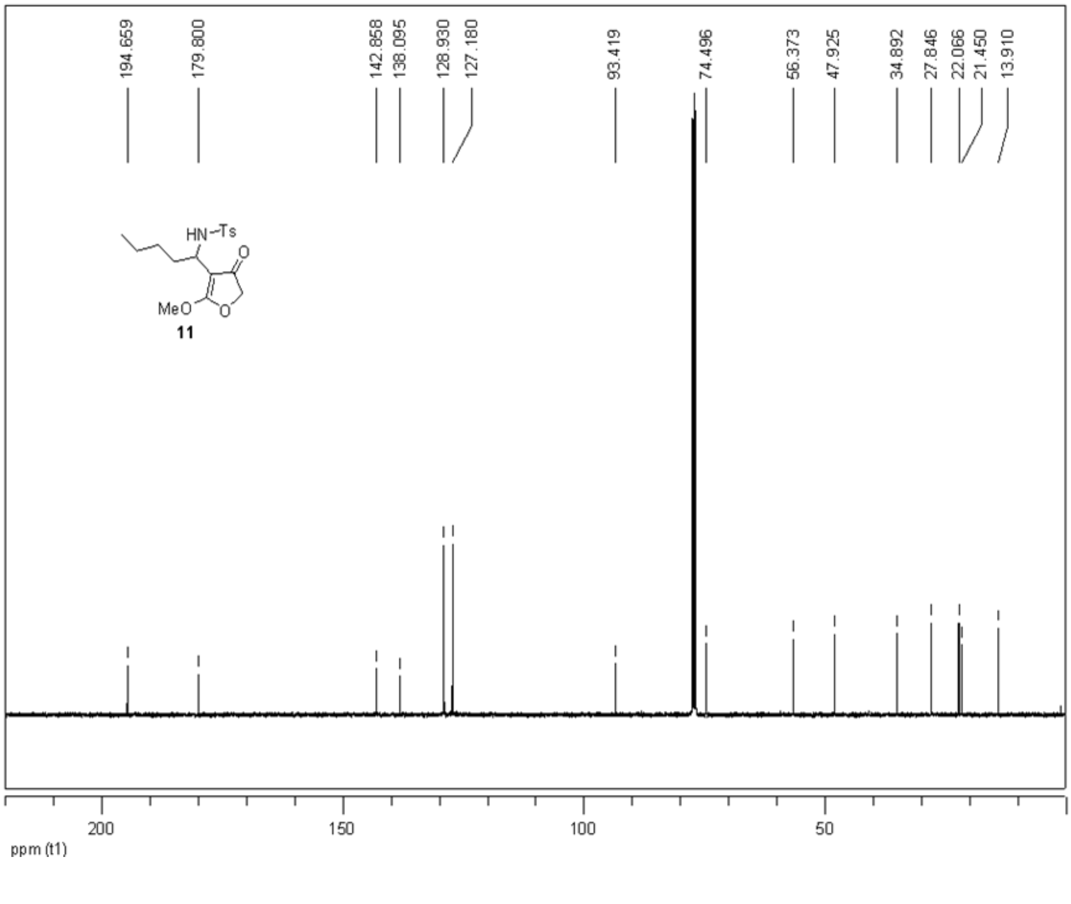

^{1}H-NMR (600 MHz)

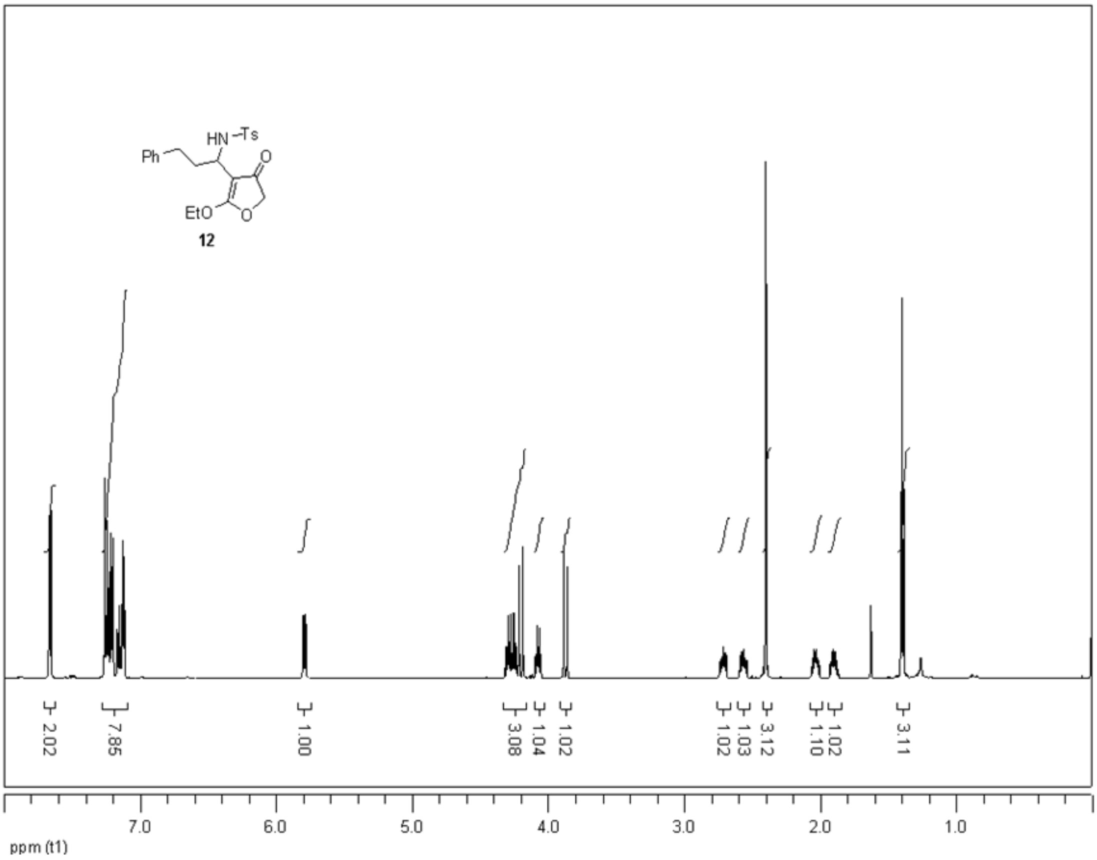

^{13}C-NMR (150 MHz)

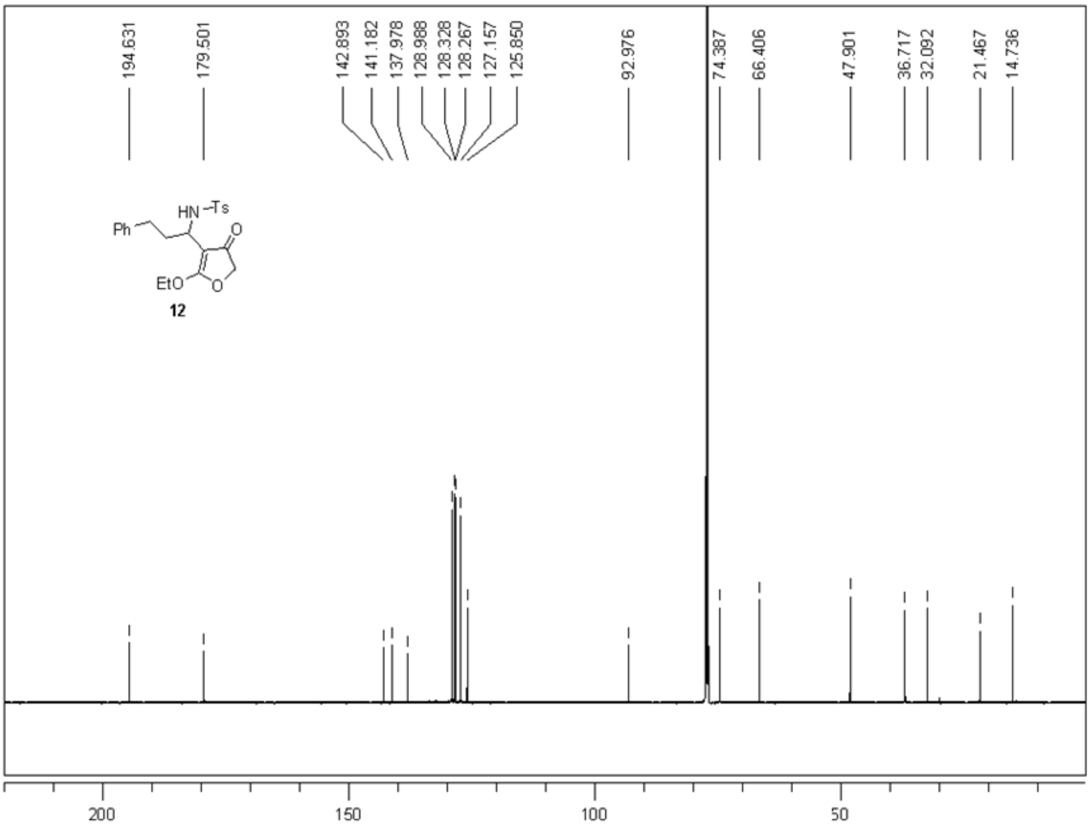

^{1}H-NMR (600 MHz)

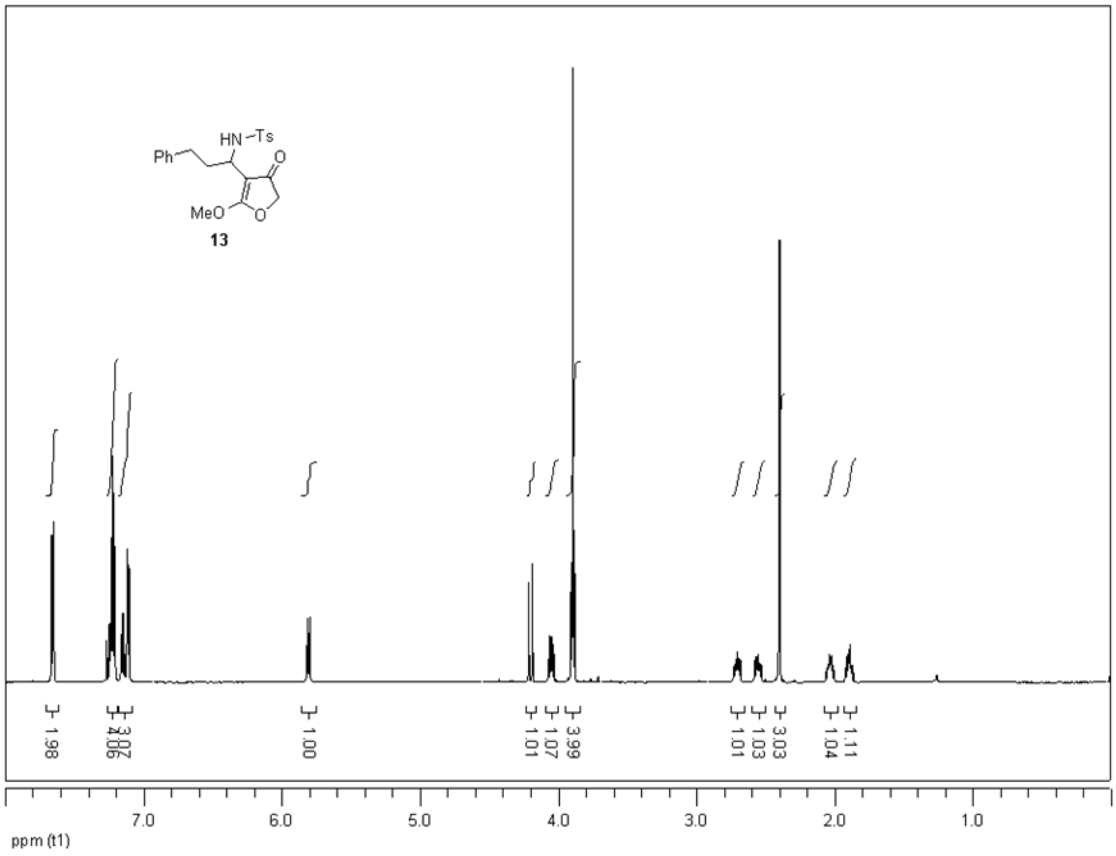

^{13}C-NMR (150 MHz)

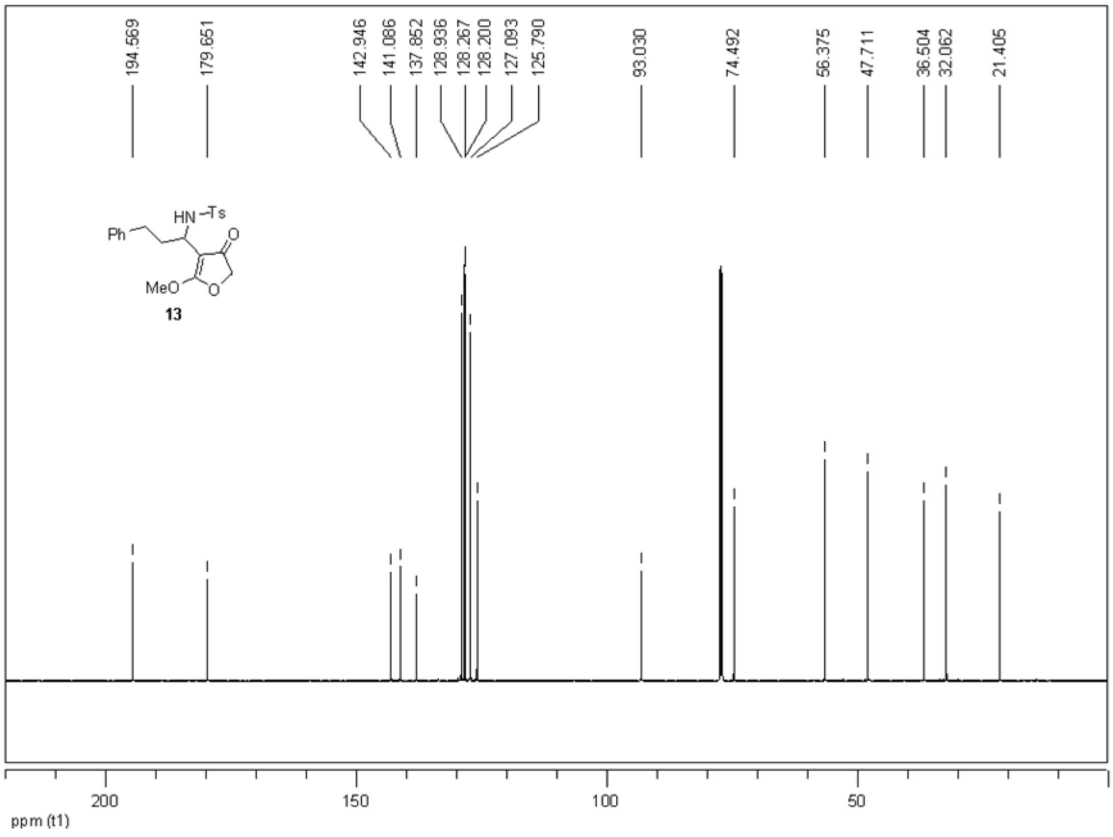

^{1}H-NMR (600 MHz)

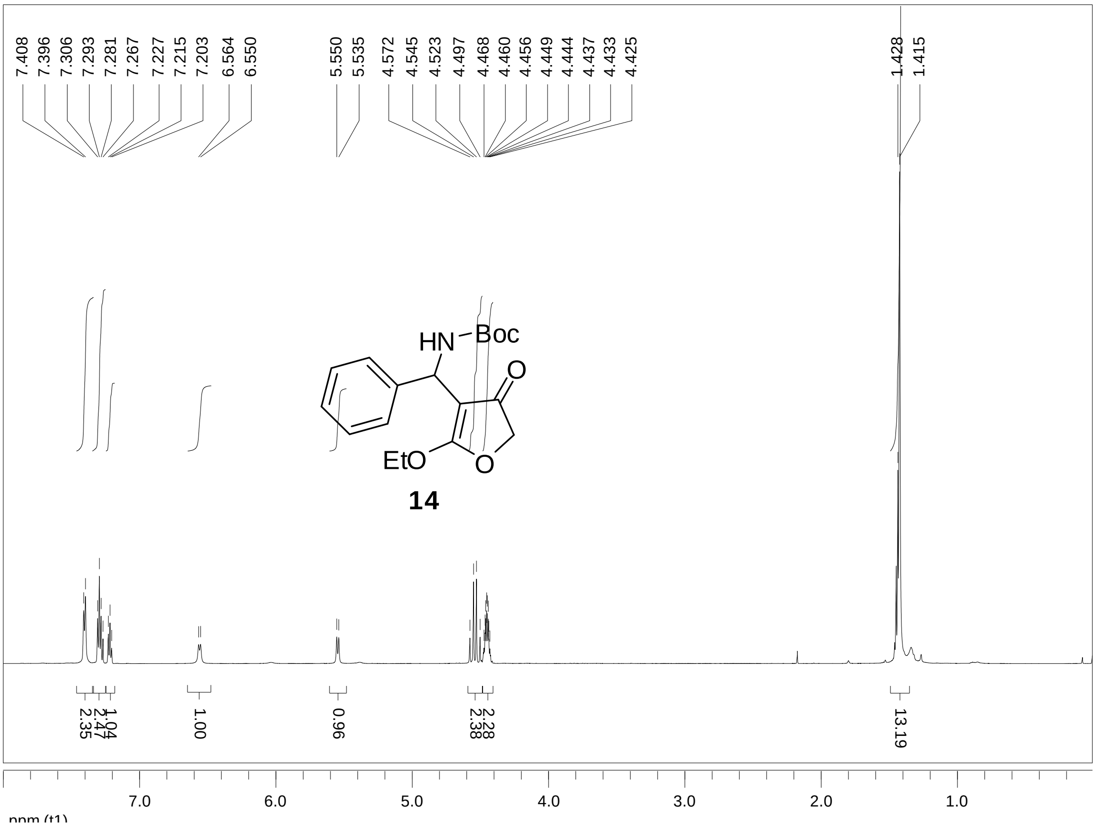

^{13}C-NMR (150 MHz)

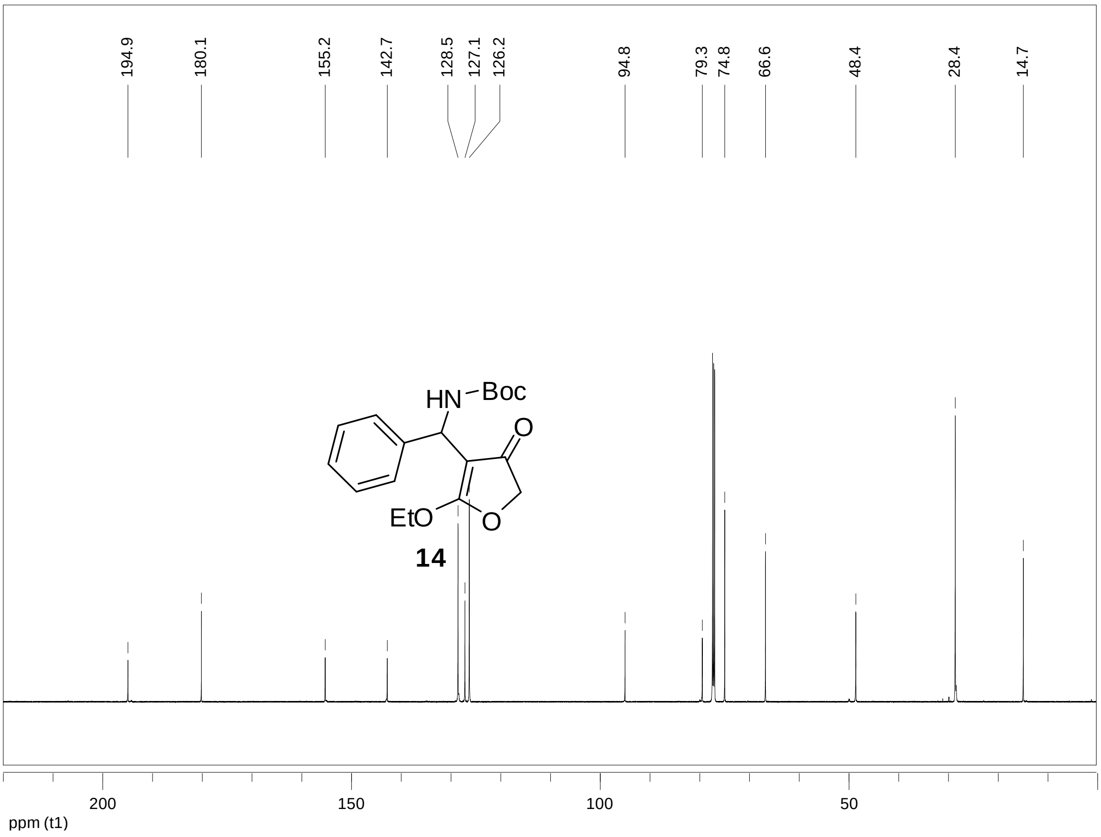

^{1}H-NMR (600 MHz)

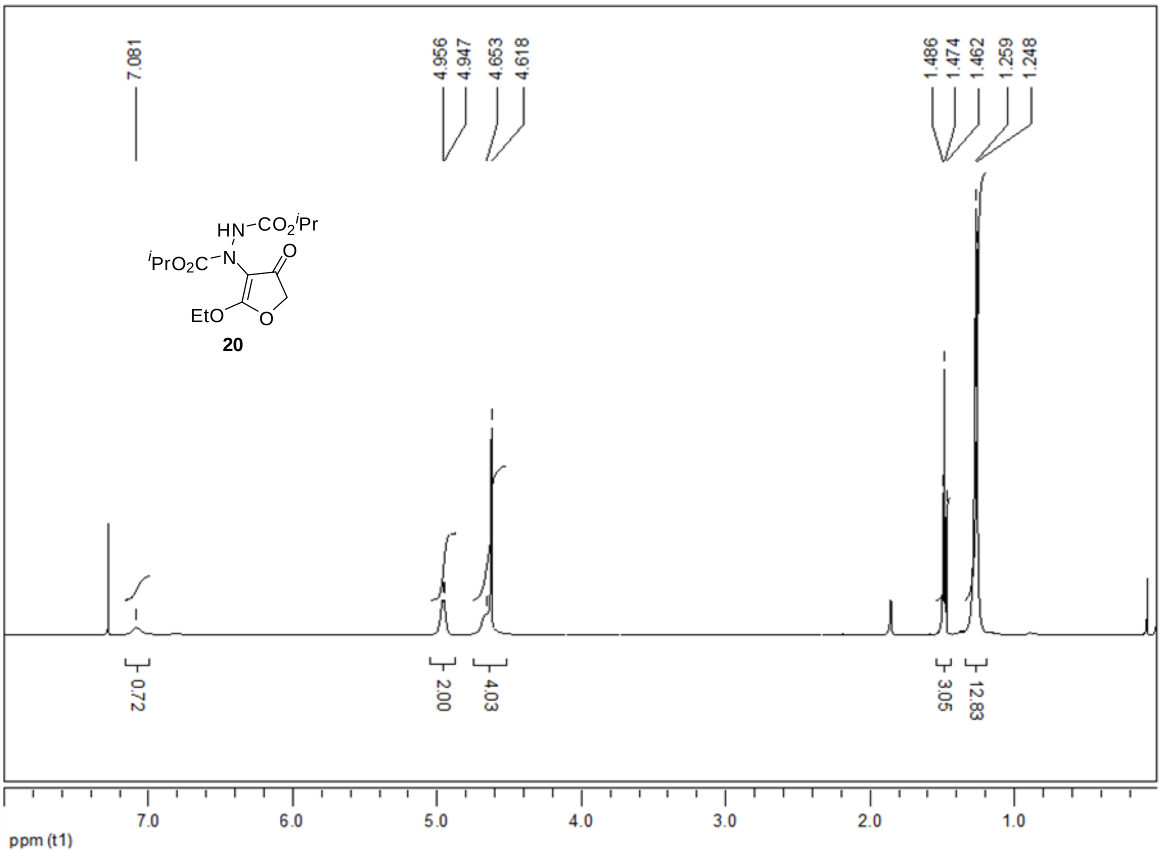

^{13}C-NMR (150 MHz)

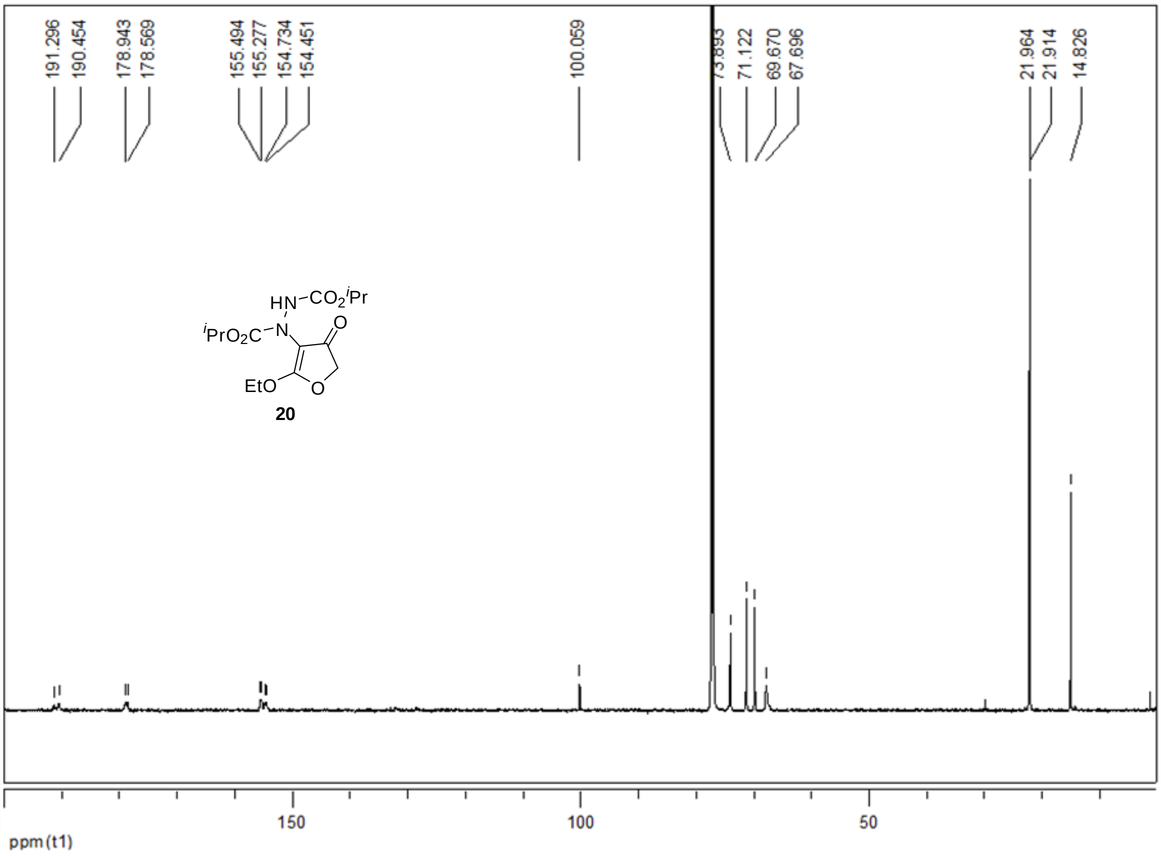

^{1}H-NMR (600 MHz)

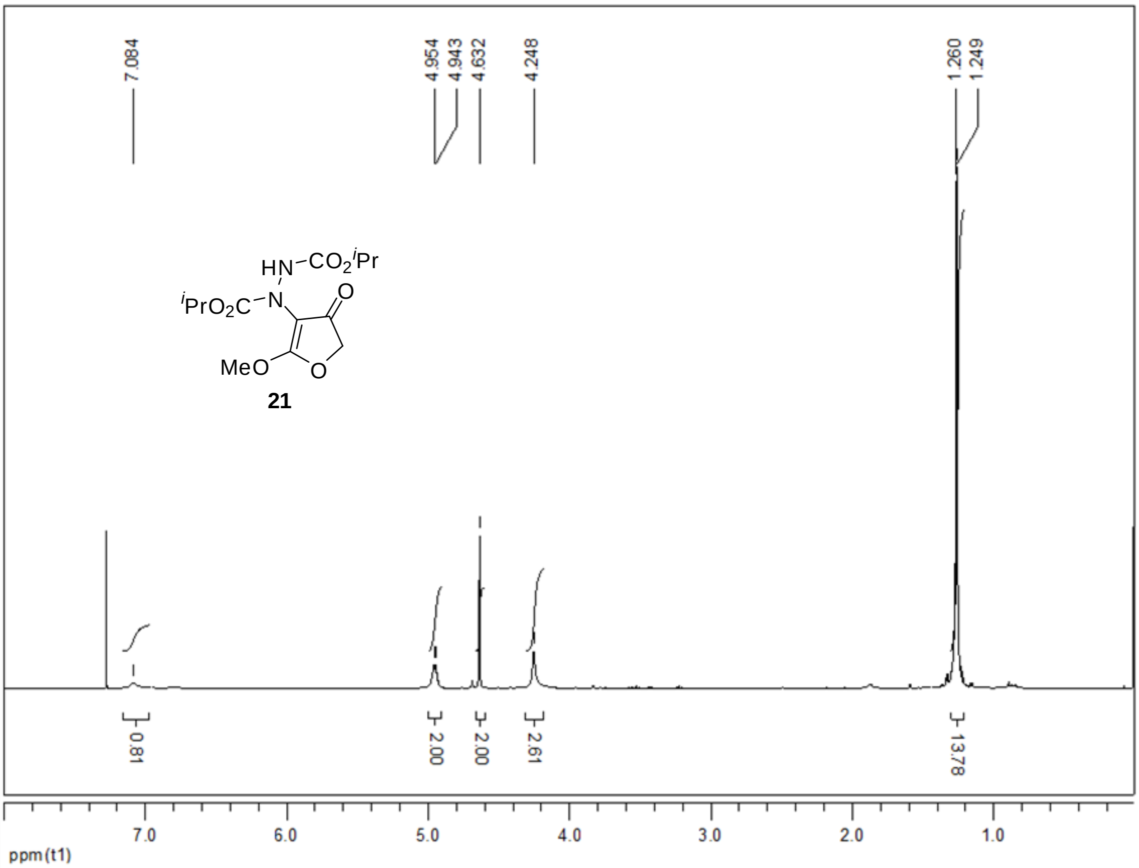

^{13}C-NMR (150 MHz)

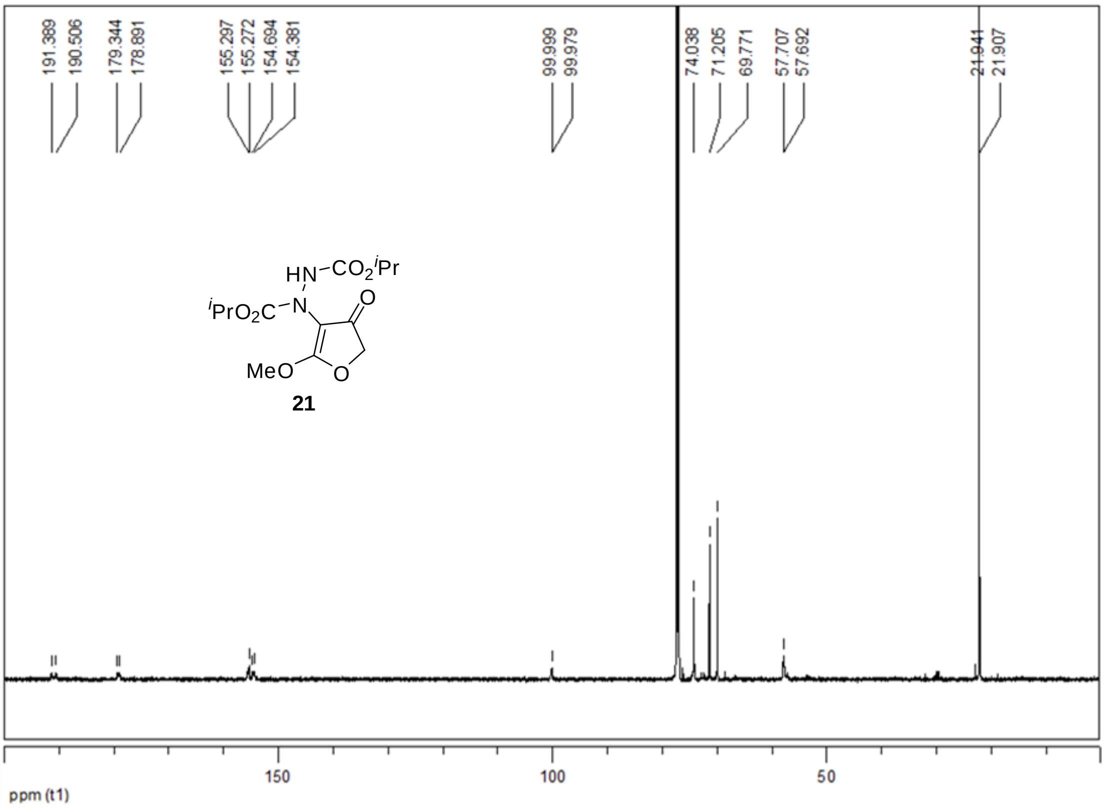

^{1}H-NMR (600 MHz)

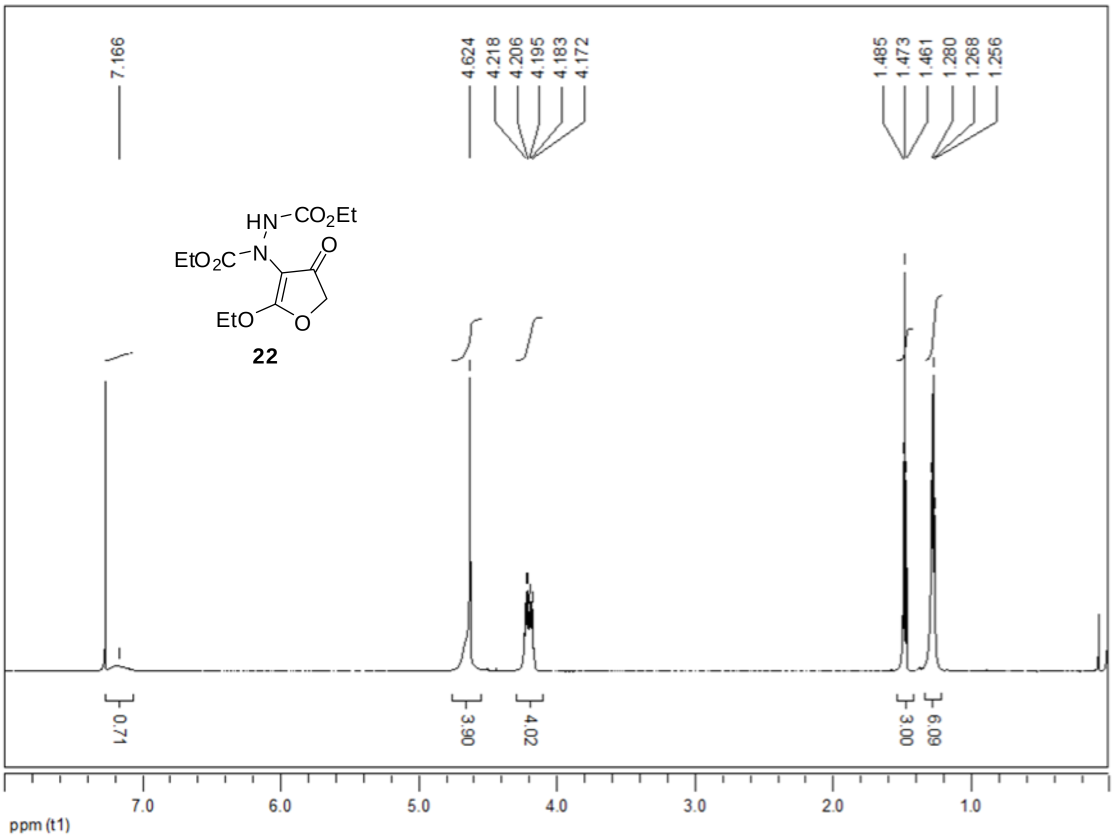

^{13}C-NMR (150 MHz)

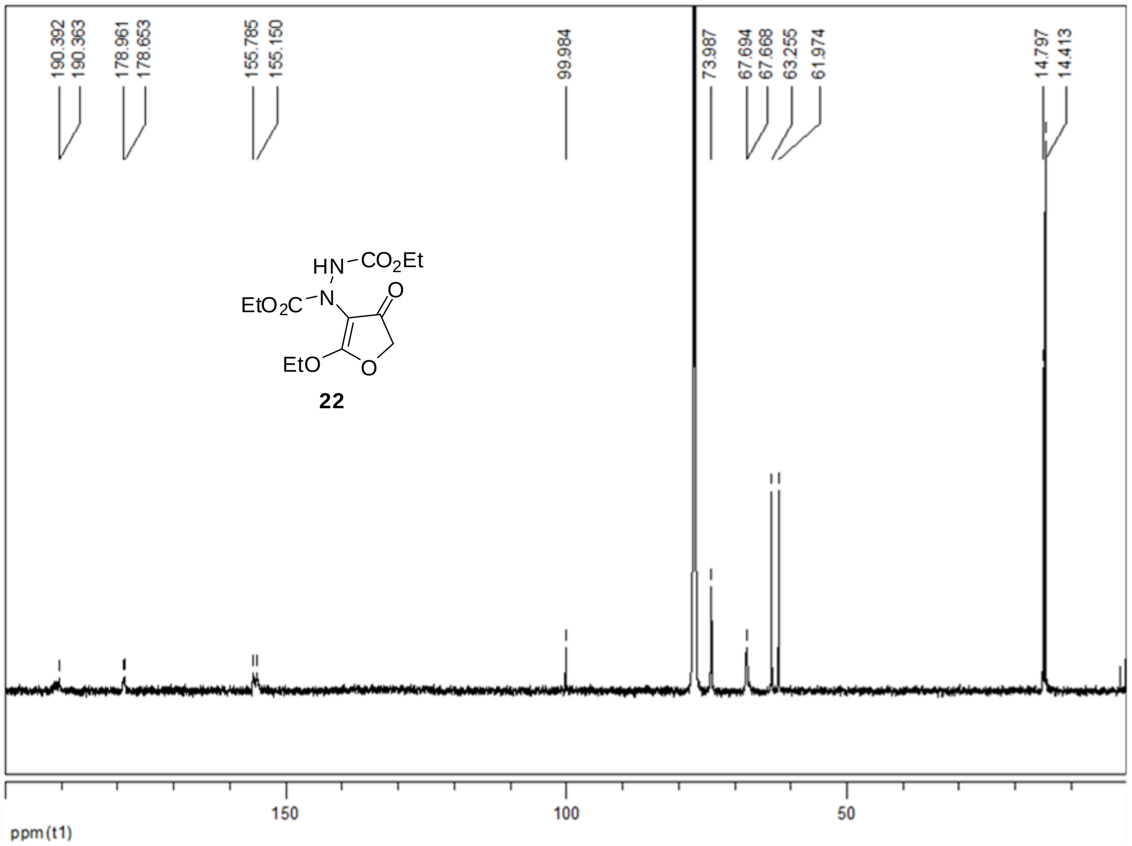

^{1}H-NMR (600 MHz)

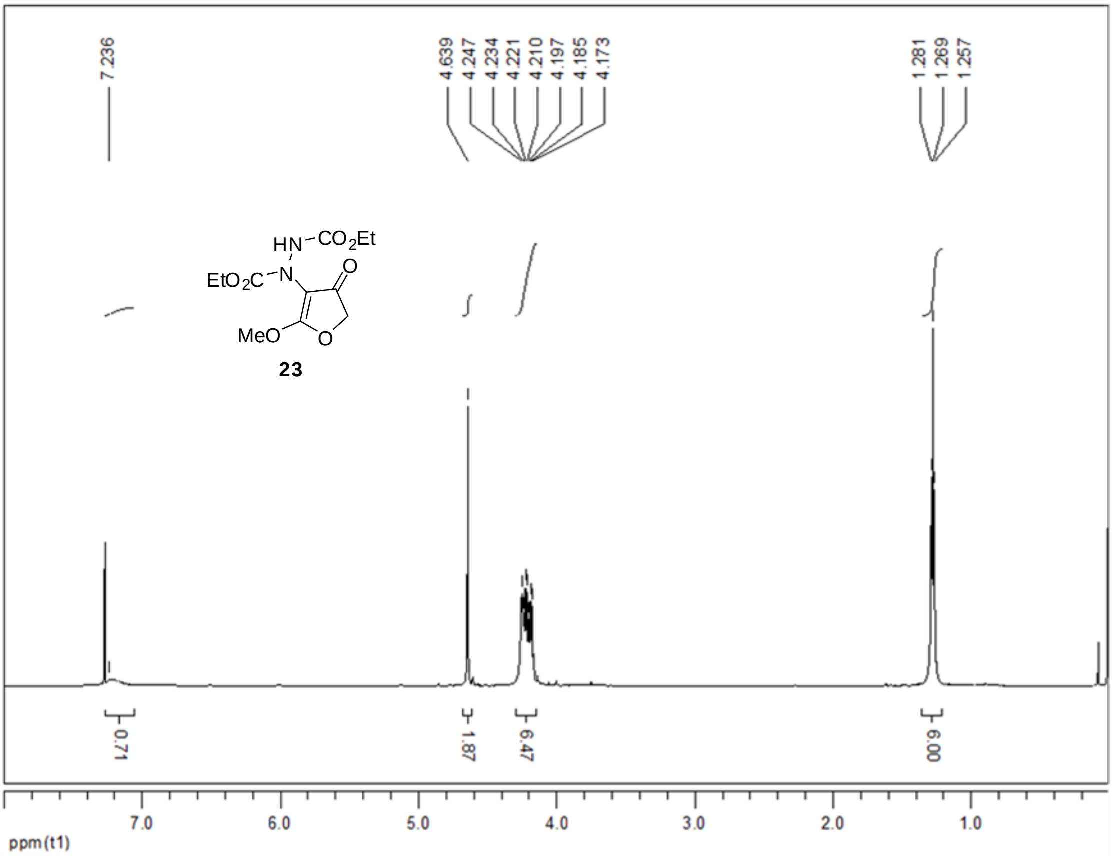

^{13}C-NMR (150 MHz)

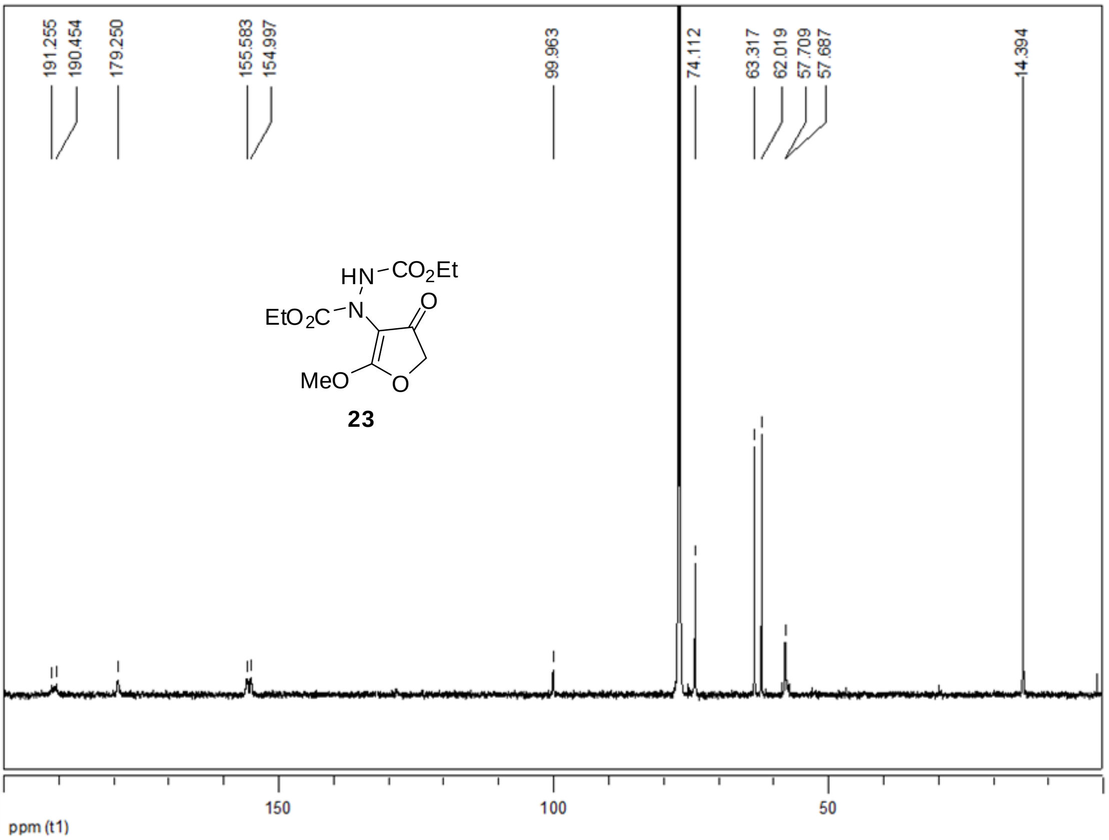

^{1}H-NMR (600 MHz)

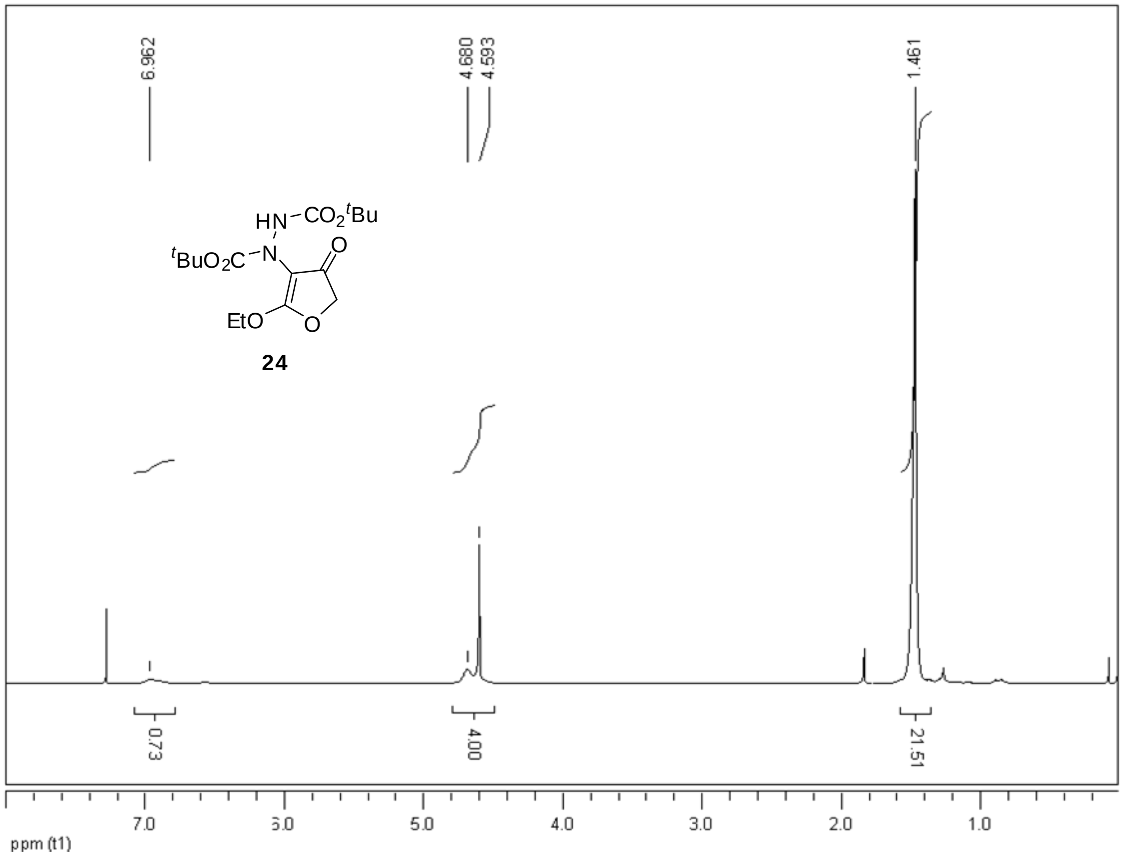

^{13}C-NMR (150 MHz)

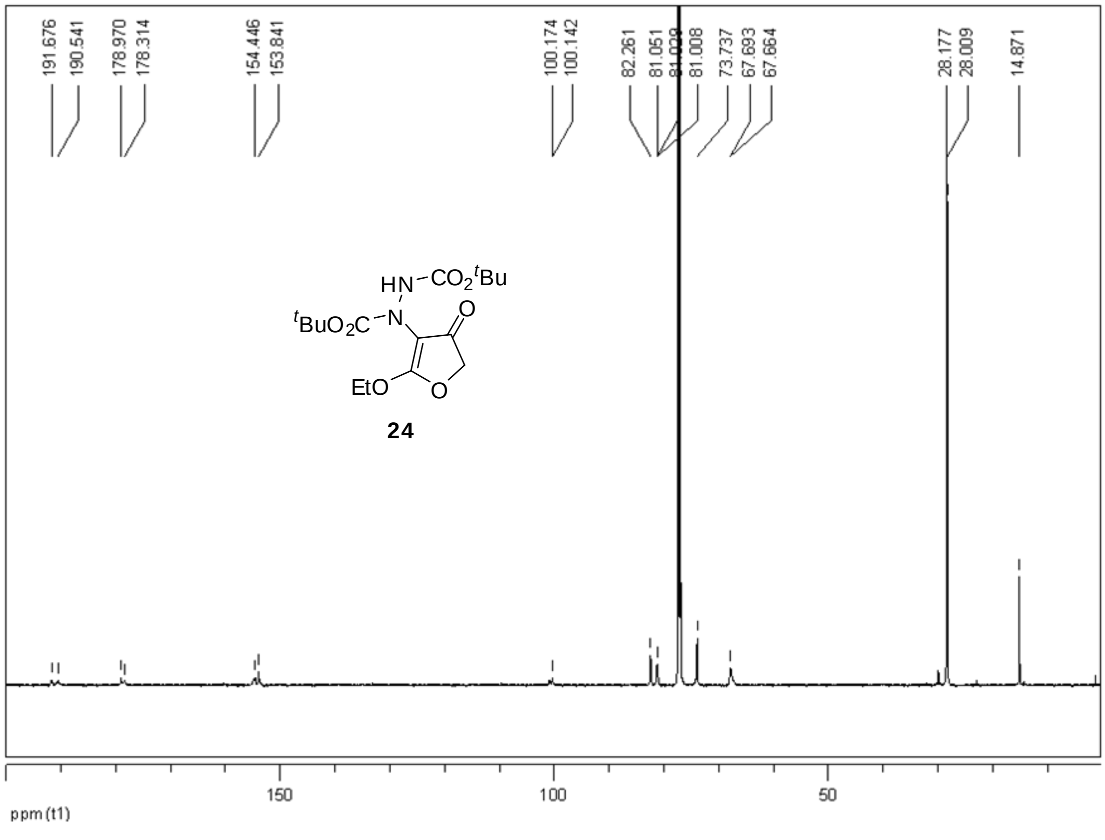

^{1}H-NMR (600 MHz)

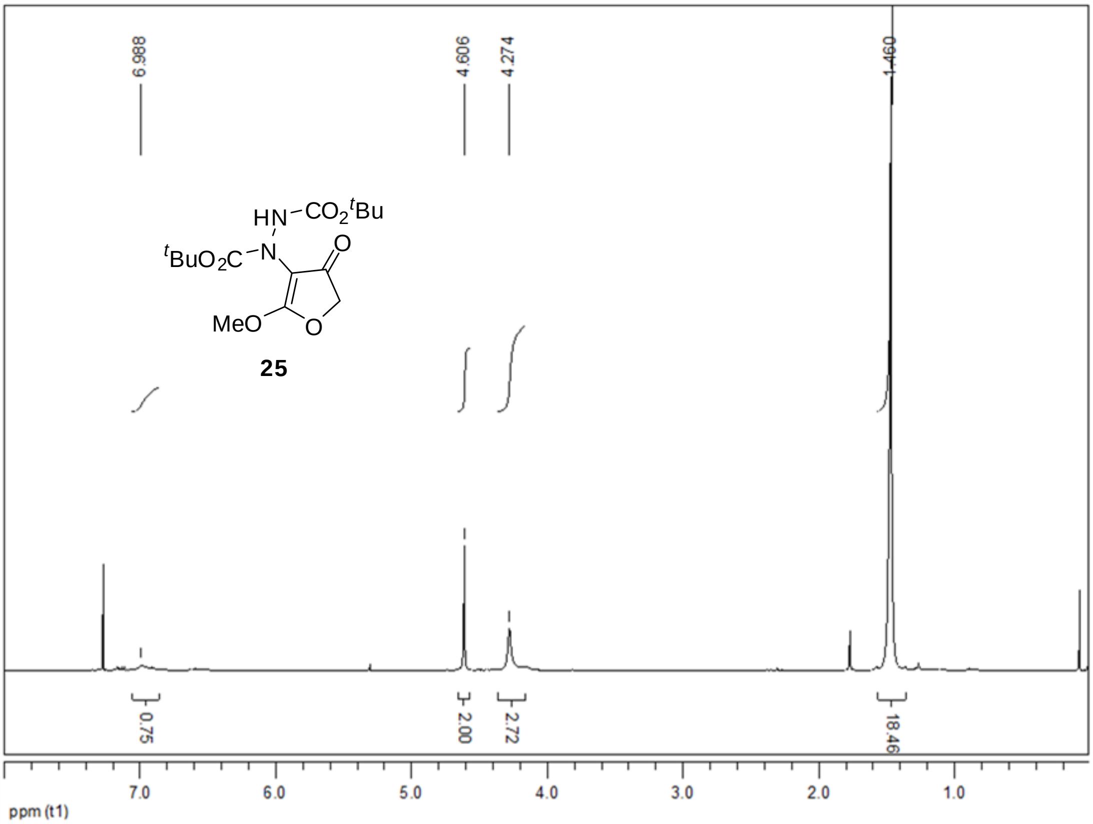

^{13}C-NMR (150 MHz)

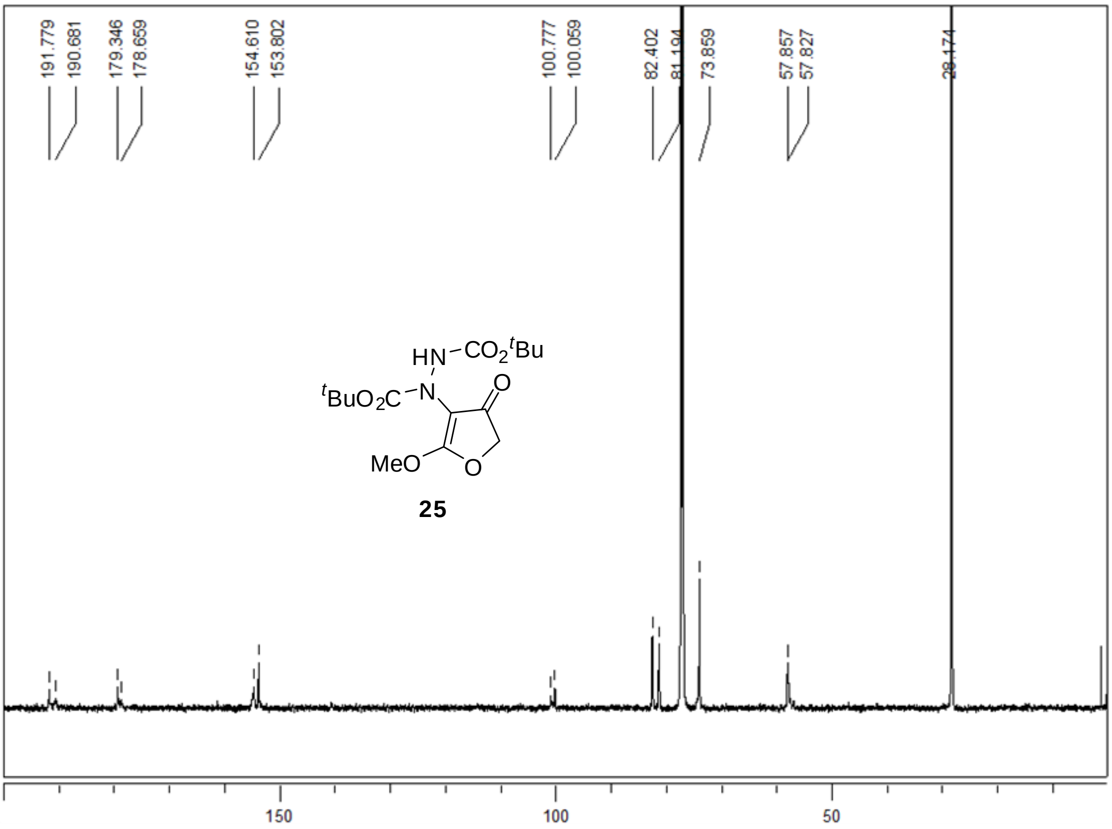

^{1}H NMR (400 MHz)

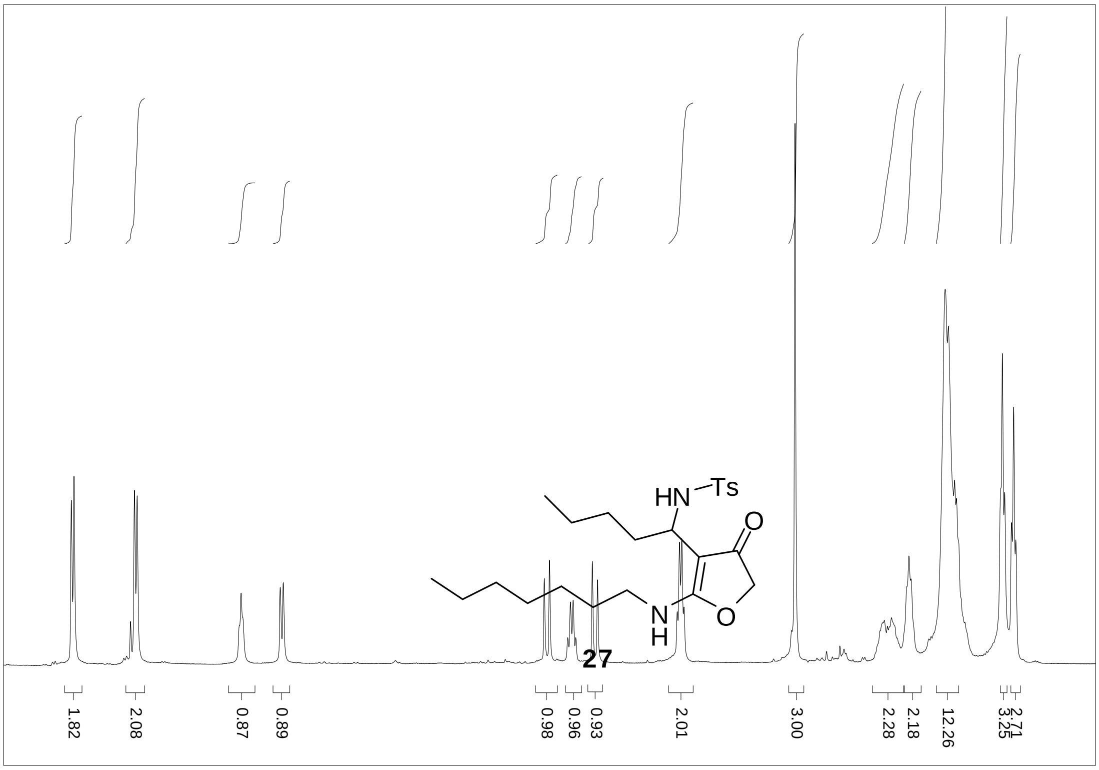

^{13}C NMR (75 MHz)

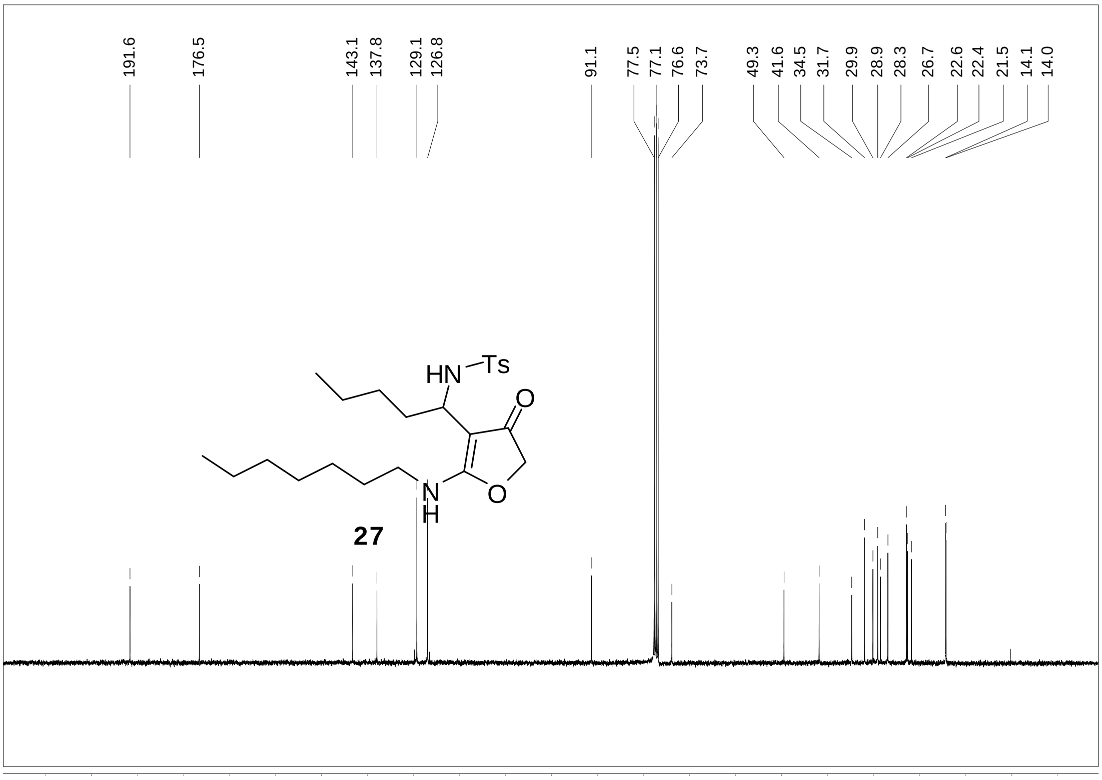

Supplement: File 1 — Experimental part and NMR spectra. [file Beilstein_J_Org_Chem-10-1462-s001.pdf]
